# Neuro-immune crosstalk in cancer: mechanisms and therapeutic implications

**DOI:** 10.1038/s41392-025-02241-8

**Published:** 2025-06-02

**Authors:** Tianyi Pu, Jiazheng Sun, Guosheng Ren, Hongzhong Li

**Affiliations:** 1 Department of Breast and Thyroid Surgery, Chongqing Key Laboratory of Molecular Oncology and Epigenetics, The First Affiliated Hospital of Chongqing Medical University, Chongqing, China; 2 Department of Pathology, Chongqing Hospital of Jiangsu Province Hospital, Chongqing, China

**Keywords:** Cancer microenvironment, Tumour immunology, Cancer microenvironment, CNS cancer, Peripheral nervous system

## Abstract

The nervous system precisely regulates physiological activities throughout the body, controlling not only muscle movement, sensory perception, cognition, and responses to external stimuli but also the immune system. In the tumor microenvironment (TME), neural components are important constituents that control the genesis, invasion, and metastasis of tumors by regulating the immune system. The nervous system modulates the tumor immune microenvironment (TIME) through localized control mechanisms (such as sensory, sympathetic, and parasympathetic innervation, as well as glial cell regulation) or via systemic adjustments, including circadian rhythm entrainment, stress modulation, and gut-brain axis regulation. To ensure their survival and proliferation, tumor cells can mimic the anti-inflammatory profiles of neuronal cells by expressing corresponding molecules to evade immune surveillance. Owing to these molecular similarities, the immune system’s targeted attack on the nervous system can lead to neurological damage, exacerbate patient conditions, and elevate mortality rates. Therefore, a detailed understanding of how the nervous and immune systems coordinate and regulate the TME can provide new perspectives and methods for the prevention and treatment of cancer. In this review, we focus on recent studies exploring the bidirectional interplay between the nervous system and tumors mediated by the immune system: how neural activity modulates tumor immunity, and conversely, how tumor-driven immune changes impact nervous system function.

## Introduction

The nervous system, comprising the central and peripheral nerve systems, along with neurotransmitters and neuropeptides, extends nerve endings throughout the body’s tissues, excluding the stratum corneum.^1^ It not only modulates a broad spectrum of physiological processes, encompassing movement, perception, cognition, and responses to external stimuli.^2^ But also it’s responsible for maintaining the stability of vital signs and the homeostasis of immune status.^3,4^ The immune system, consisting of immune organs, cells, molecules, and lymphatic vessels, is similarly widespread and tasked with the identification and elimination of pathogens and tumor cells through antibody production and immune cell activation. The interplay between the nervous and immune systems is essential for homeostatic maintenance, which is pivotal in tumor prevention and control.^5^ While TME is typically considered the local environment surrounding tumor cells, which includes a complex ecosystem composed of tumor cells, immune cells, fibroblasts, endothelial cells, extracellular matrix (ECM), and secreted factors.^6–8^ Often overlooked, neural components within the TME are a critical part of this ecosystem.^9^ In fact, nerve endings extend into the TME,^10^ along with leukocytes that are present there, regulate the growth, invasion, and metastasis of tumor cells.^11–13^ Early in tumor development, the dysfunction of the nervous and immune systems can lead to a failure to promptly eliminate the tumor, providing it with an opportunity to continue growing. During the growth of the tumor, the nervous system may act as a sanctuary,^14^ and cytotoxic T cells may become exhausted.^15,16^ These factors can contribute to uncontrollable tumor growth. However, the precise mechanisms by which the nervous system governs and regulates the immune system are not yet fully understood in TME. This field has attracted growing attention in recent years.

The nervous system can sense the immune status within TME and relay this information to the brain, which integrates the incoming data and acts back on TME, forming a comprehensive neural circuit.^17^ Within this circuit, the sensory nerve and the vagus nerve are responsible for gathering immune information from TME and conveying it to the brain. After integrating the information, the brain can feed back to the immune system^18^ that can be activated to regulate leukocyte trafficking and function. This pathogenesis can influence the effectiveness of anti-cancer immunity or immunotherapeutic strategies.^19^ The brain can also regulate the immune status of tissues through the vagus or sympathetic nerves,^20^ such as limiting the secretion of inflammatory factors in experimental lipopolysaccharide (LPS)-induced sepsis.^1^ Furthermore, the brain can exert regulatory effects on TME through the hypothalamic–pituitary–adrenal (HPA) axis.^21,22^ The circadian rhythm and the gut-brain axis are also integral components in the nervous system’s regulation of the TME. The suprachiasmatic nucleus (SCN), situated in the anterior hypothalamus, serves as the pacemaker of rhythm, coordinating the rhythm of peripheral tissues through genetic and protein networks, ensuring harmonious physiological functioning.^23^ Current study is increasingly focusing on the gut-brain axis, where the intestinal tract, enriched with neural and immune components, plays a significant role in tumor immunity through its interactions with the gut microbiota.^24^ Intriguingly, tumors can also exert substantial influence on the nervous system through the immune system, indicating a bidirectional relationship.^25^

Therefore, a detailed understanding of the mechanisms by which the nervous and immune systems interact in cancer is extremely important for both the prevention and treatment of cancer. In this review, we summarize the interplay between the nervous and the immune systems in the TME.

## Neuroregulation of the immune system

**Fig. 1:**
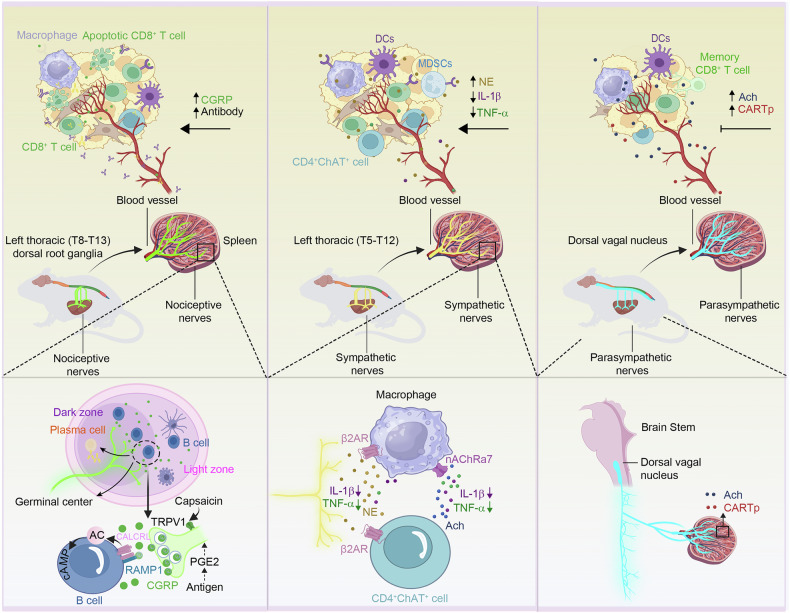
The neural innervation of the spleen. Spleen-innervating nociceptors, which originate from left T8 to T13 dorsal root ganglia and innervate the spleen along blood vessels to reach B cell zones, where nociceptor-derived CGRP promotes germinal center responses via the CALCRL-RAMP1 receptor on B cells. Activating these nociceptors with capsaicin enhances proliferation of germinal centers and plasma cells (PCs), increases secretion of antibody, improvement of humoral immunity, and host defense. The increased antibodies and CGRP entering the bloodstream may lead to elevated levels of antibodies in TME, thereby potentially influencing antitumor immunity. The splenic sympathetic nerves mainly originate from the T5 to T12 segments of the thoracic spinal cord, enter the spleen along the blood vessels, and innervate the spleen. The sympathetic nervous system releases NE, which interacts with the β2-AR present on macrophages. This interaction results in a reduced secretion of IL-1β and TNF-α. Additionally, NE influences ChAT^+^ CD4^+^ T cells, prompting them to release ACh. ACh binds to α7nAChR on macrophages, further contributing to the suppression of IL-1β and TNF-α release. Consequently, the diminished levels of these pro-inflammatory mediators in the bloodstream may modulate the body’s antitumor immune response as well as NE in the TME. The existence of parasympathetic innervation to the spleen is controversial, however, a recent study shows that there are parasympathetic nerves directly projected to the spleen. The presence of Ach and cocaine- and amphetamine-regulated transcript peptide (CARTp) within splenic tissues has been documented. Alterations in the blood concentrations of ACh and CARTp may modulate antitumor immunity. This figure was created with BioRender (https://biorender.com/)

The nervous and immune systems are often described and studied as distinct entities,^26^ however, over the past few decades, numerous studies have reported that hematopoietic and lymphatic organs have abundant neural innervation, which greatly facilitates the exchange between nerves and leukocytes.^27–29^ Calcitonin gene-related peptide (CGRP) sensory and sympathetic nerve innervation have been confirmed in lymph nodes,^30–34^ spleen^35–37^ (Fig. 1), bone marrow,^38–41^, and thymus.^42,43^ CGRP is present in the thymus, although there is no direct evidence to confirm its release by CGRP neurons.^42^ Adrenergic nerve controls blood vessels or directly modulates the function of lymph nodes through the norepinephrine (NE)/β1 and β2-adrenergic receptor axis.^44–50^ A Study has reported that the brain’s reward system can diminish the immunosuppressive function of bone marrow-derived suppressive cells (MDSCs) in the bone marrow by activating the sympathetic nervous system, thereby slowing tumor growth.^51^ For a long time, it has been uncertain whether the immune organs are innervated by the parasympathetic nervous system,^36,39,43,52–54^ but recently, a study shows that the spleen has direct parasympathetic innervation^55^ (Fig. 1).

The current understanding of the neural center that regulates the immune system is not yet clear. Some studies have found that when inflammation occurs in peripheral tissues, a group of cells in the insular cortex of the brain (InsCtx neurons) are activated.^56^ Inhibiting the InsCtx neurons can improve peripheral inflammation. In the absence of peripheral inflammation, stimulating the InsCtx neurons can reproduce the effects of peripheral inflammation. These cells project to areas controlled by the autonomic nervous system, such as the dorsal motor nucleus of the vagus (DMV) and the rostral ventrolateral medulla (RVLM).^56^

Studies on neuro-immune circuits are still relatively limited. It was discovered that the vagus nerve, upon sensing peripheral inflammation, activates the caudal Nucleus of the Solitary Tract (cNST) and the Area Postrema (AP), suggesting that these may be the sensory nuclei for immune regulation.^18^ Another study reported that neurons in the central nucleus of the amygdala (CeA) and the paraventricular nucleus (PVN), which contain corticotropin-releasing hormone (CRH), are connected to the splenic nerve via the sympathetic nervous system.^57^ It has been demonstrated that motor circuits rapidly mobilize neutrophils from bone marrow to peripheral tissues by neutrophil-attracting chemokines derived from skeletal muscle. In contrast, PVN controlled the migration of monocytes and lymphocytes from secondary lymphoid organs and blood to the bone marrow via direct, cell-intrinsic glucocorticoid (GC) signaling.^58^ A similar response has been observed in breast cancer, where tumor burden leads to widespread anxiety in mice. The activation of CRH neurons in CeA led to the proliferation of sympathetic fibers in the breast cancer TME, promoting tumor growth.^59^ The reduction of sympathetic nerves in TME and the slowing of tumor growth was achieved by pharmacological or genetic blockade of the CRH neurons in the CeA to the lateral paragigantocellular nucleus (LPGi) circuit. Furthermore, the use of alprazolam, an anti-anxiety medication, hindered the growth of breast cancer.^59,60^

**Fig. 2:**
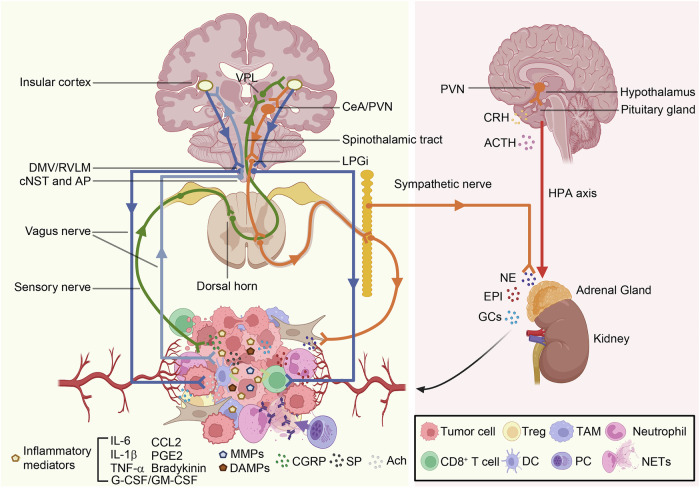
Neuro-immune-cancer circuits. Sensory nerves detect signal molecules (such as inflammatory mediators, MMPs, DAMPs) within TIME, leading to the release of CGRP and transmission of signals to the dorsal horn of the spinal cord. Subsequently, the signals are relayed through the spinothalamic tract to the ventral nucleus of the thalamus (VPL), where the signals are further conveyed to neurons within InsCtx. The response signals are then transmitted to CeA and PVN from InsCtx, followed by projections to LPGi, and then transmitted to sympathetic nerves, which modulate the TIME through NE. Vagus nerves can also detect signal molecules within TIME, and transmit these molecules to the cNST and AP. The signals are then relayed to neurons within InsCtx, and the response signals are transmitted to DMV and RVLM, ultimately returning to the TIME via the vagus nerves. In addition, sensory nerves detect signal molecules within the TIME, leading to the release of CGRP and signal transmission to the dorsal horn of the spinal cord, followed by transmission through the spinothalamic tract to VPL. The signals continue to neurons within InsCtx, subsequently the response signals are transmitted to DMV/RVLM, and then relayed to vagus nerves that modulate TIME via Ach. Furthermore, vagus nerves detect signal molecules within TIME, transmit signals to cNST and AP, and then relayed to neurons within InsCtx, followed by the transmission of response signals to the CeA/PVN, leading to increased activity in LPGi. Ultimately, the signals are transmitted to sympathetic nerves that modulate TIME through NE. Moreover, PVN is a key brain region in activating the HPA axis, controlling immune responses through the release of GCs and CAs from the adrenal glands. This figure was created with BioRender (https://biorender.com/)

Based on the aforementioned studies,^18,56–59^ we can summarize several neural-immune-cancer circuits (Fig. 2). They follow the neural reflex pattern of sense-neural center-effect. Afferent nerves detect inflammatory signals within TME,^18^ conveying them to the neural center,^56^ which then integrates this information and feeds back to TME via efferent nerves.^59,60^ However, in different tissues, the pathways of neuro-immune-cancer circuitry may vary because the distribution of nerves in the skin, muscles, and viscera is distinct. Consequently, the neuro-immune regulation of tumors in different locations may also differ. These neuro-immune-cancer circuits require more experimental validation.

## Neuro-immune regulation of central nervous system (CNS) tumors

### Immune surveillance in the meninges

The traditional view holds that the CNS is an immunologically privileged organ, due to the presence of the blood–brain barrier (BBB) and an absence of lymphatic vessels.^61^ However, a recent study discovered the existence of functional lymphatic vessels within the CNS. These vessels are located near the dural sinuses, express lymphatic endothelial cell markers, and can carry cerebrospinal fluid (CSF) and immune cells. They are closely related to the circulation of CSF and are connected to the deep cervical lymph nodes.^62^ CSF exchanges with the brain’s interstitial fluid, aiding in the clearance of metabolic waste from the brain, such as amyloid-beta (Aβ). The CSF, carrying antigens and waste, circulates through the ventricles, cisterns, the central canal, and the subarachnoid space, with the lymphatic vessels responsible for collecting CSF and large molecular substances, including antigens, transporting them to the deep cervical lymph nodes to activate an immune response.^63^

Immune cells of the CNS originate not only from the blood supply of the heart but also from the skull marrow. The skull marrow produces a variety of immunocytes, including B cells, monocytes, and neutrophils that can migrate to the meninges through direct channels between the skull and the meninges, and even enter the CNS parenchyma in pathological states.^64^ The meninges contain diverse immunocytes, including macrophages, dendritic cells (DCs), T cells, and B cells, which can directly contact CSF, monitor, and respond to pathological changes in the CNS. Researchers have discovered “arachnoid capillary exits” points, which allow the exchange of CSF and molecules between the subarachnoid space and the dura mater.^63^ Immune cells in the meninges can also support humoral immunity of the meninges through organized lymphoid-like structures (dural-associated lymphoid tissues, DALT). Particularly, the rostral-rhinal venolymphatic hub structure, which surrounds the anterior part of the sinuses and the nasal confluence, includes lymphatic vessels that can sample antigens and rapidly support humoral immune responses.^65^ When the CNS is infected or damaged, the levels of inflammatory factors and antigens in the CSF will rise and these changes can activate immune cells in the meninges which can migrate to the lymph nodes through the lymphatic vessel system, initiating a broader immune response, such as the production of antibodies and activation of T cells.^65^

### Immune surveillance in the brain

The immune functions within the brain are regulated by microglia. Microglia can be polarized into either a pro-inflammatory (M1-type microglia) or an anti-inflammatory and tissue repair (M2-type microglia) state.^66^ In response to pathogens, microglia activate T cells and secrete inflammatory factors, including TNF-α, IL-6, and IL-1β.^67^ Subsequently, to mitigate this response, T cells release cytokines such as IL-4 and IL-13, which help to shift microglia from the M1 to the M2 phenotype.^68^ In the immune system, helper T (Th) cells are T lymphocytes that produce cytokines. Th1 cells generate pro-inflammatory cytokines, while Th2 cells produce anti-inflammatory factors, including IL-10.^69^

### Immune cells-glioma crosstalk

Tumors originating from the CNS encompass a spectrum of neoplasms, among which gliomas are the most prevalent. Gliomas arise from the glial cell lineage, specifically from astrocytes, oligodendrocytes, and ependymal cells, which are distinct glial cell types of the CNS.^70–72^ Utilizing gliomas as a paradigm, we delve into the intricate interplay of neuro-immune-cancer crosstalk, a dynamic and pivotal area of research within the scientific community.

**Fig. 3:**
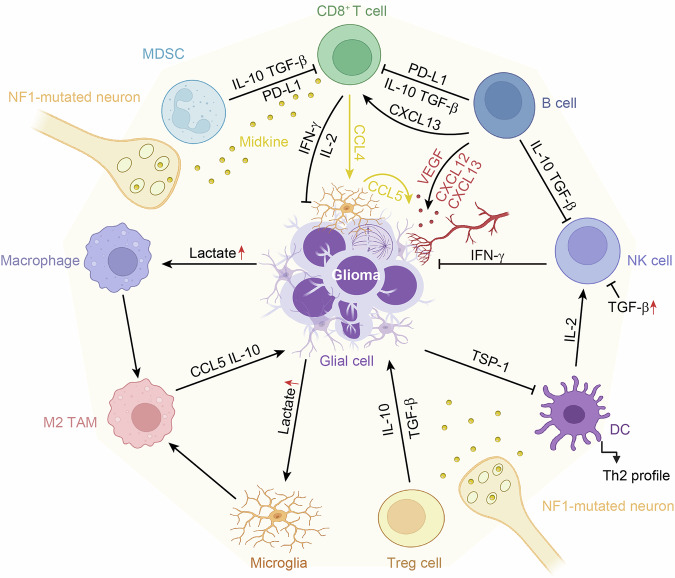
Neuro-immune crosstalk in CNS tumors. Glioma-associated microglia and macrophages are attracted to the glioma. The metabolism of gliomas can produce a large amount of lactate, which promotes the transformation of microglia and macrophages into M2-type TAMs that suppress antitumor immunity and support tumor growth. Activated microglia secrete IL-10 and CCL5, supporting tumor formation and growth. Midkine activates CD8^+^ T cells, which produce CCL4, stimulating microglia to produce CCL5, CCL5 is crucial for the stem cells of low-grade glioma to survive and grow. IL-2 and IFN-γ boost T and NK cell growth/activation, enhancing immune surveillance of cancer cells. The inhibition of NK cell function in brain tumors may be partly due to the increased levels of TGF-β in the plasma. Elevated Tregs in gliomas produce IL-10 and TGF-β, promoting tumor growth. MDSCs adapt to the TME and suppress T cells via molecules like PD-L1, IL-10, and TGF-β. B lymphocytes express PD-L1, which engages with the PD-1 on T cells, resulting in T cell exhaustion and facilitating glioma evasion. Additionally, these B lymphocytes generate IL-10 and TGF-β, which dampen the activity of immune cells, including T cells and NK cells. Furthermore, they release VEGF, CXCL12, and CXCL13, promoting the formation of new blood vessels to nourish the glioma. Glioma cells are capable of upregulating thrombospondin-1 in DCs, which inhibits their maturation and biases cytokine secretion toward a Th2 profile, thereby potentially inducing a state of immunosuppression. This figure was created with BioRender (https://biorender.com/)

During gliomagenesis, the BBB is compromised, allowing circulating T cells, B cells, macrophages, and MDSCs to cross the BBB.^73^ The reciprocal signaling between immunocytes and glioma cells establishes an immunosuppressive microenvironment to foster tumor growth^74^ (Fig. 3). From a 3D spatial perspective, different regions of the tumor may have varying densities of immune cells, such as T cells, B cells, natural killer (NK) cells, DCs, microglia, and macrophages, with some areas being densely populated and others relatively sparse.^75^ CD4^+^ and CD8^+^ T cells show different distribution patterns in various regions of the tumor. TME contains immunosuppressive cells, such as regulatory T cells (Tregs) and tumor-associated macrophages (TAMs), which are unevenly distributed across the tumor, affecting the immune response. Immune cells display different activation or exhaustion statuses in various regions of the tumor. For instance, T cells in the central region of the tumor are exhausted due to hypoxia and nutrient deficiency, while T cells at the tumor periphery are more active.^75^

Glioma-associated microglia and macrophages are attracted to the tumor,^76^ where they are polarized into M2-like cells that suppress tumor immunity and support tumor growth.^77,78^ Activated microglia secrete cytokines and growth factors that promote the growth and angiogenesis of tumor cells, such as IL-10, epidermal growth factor (EGF), and vascular endothelial growth factor (VEGF).^76^ Microglia can also express the chemokine CCL5, which facilitates the proliferation and survival of oligodendrocyte precursor cells (OPCs), thereby supporting tumor formation and growth. Activated T cells secrete soluble factors that stimulate microglia to express CCL5, thus providing a supportive microenvironment for the engraftment of OPCs. In mice with a genetic deletion of *Ccl5*, OPCs fail to form tumors. These findings suggest that interventions targeting the interaction between T cells and microglia/macrophages could offer new strategies for the treatment of low-grade gliomas.^79^ Lactate produced by glioma cells can induce the polarization of TAMs toward the M2 phenotype. High-mobility group box 1 secreted by TAMs can promote the proliferation, migration, invasion, and mesenchymal transition of glioma cells.^80^ Lactate dehydrogenase A (LDHA) modulates the behavior of macrophages by regulating the metabolism and secretory signals of tumor cells, thereby promoting the progression of glioblastoma. Targeting LDHA or its downstream signaling pathways may provide new therapeutic strategies for glioblastoma.^81^ Although microglia are important in tumor immune suppression, recent studies show that macrophages from blood monocytes (MDMs) are the main immune suppressors in glioblastoma multiforme (GBM), rather than microglia. MDMs in GBM have high glycolysis linked to their suppressive role. GBM factors boost MDM glycolysis, lactate production, and IL-10, which supports T cell suppression through histone lactylation. GBM also activates the PERK-ATF4 pathway in MDMs, increasing GLUT1 for more glycolysis. Removing PERK in MDMs can stop histone lactylation, increase T cells in tumors, slow tumor growth, and enhance GBM treatment with immunotherapy. Targeting PERK to counteract histone lactylation could improve immunotherapy for GBM.^82^ Chronic stress can advance GBM by reducing immune cells in tumors, especially M1 macrophages and CD8^+^ T cells. It hinders the secretion of CCL3/4, affecting M1 macrophage recruitment. But adding CCL3 can improve the immunosuppressive environment and fight GBM progression caused by stress. It has been confirmed that CCL3 boosts the efficacy of immune checkpoint therapy in GBM.^83^ A subset of TAMs, termed lipid-laden macrophages, exhibit an immunosuppressive phenotype and meet the high metabolic demands of GBM by engulfing cholesterol-rich myelin debris, accumulating cholesterol, and directly transferring lipids to cancer cells to nourish them.^84^ In brain metastasis cancer, meningeal macrophages can secrete glial cell line-derived neurotrophic factor (GDNF) to aid breast cancer cells in surviving within the meninges, thereby promoting metastasis, depending on the binding of integrin α6 to laminin.^85^

NK cells are a component of the innate immune system, capable of killing tumor cells without prior sensitization. IL-2 and IFN-γ enhance the surveillance of cancer cells by the immune system by promoting the growth and activation of T cells and NK cells.^86–89^ In contrast, the downregulation of NK cell function in gliomas may be partly due to the increased levels of TGF-β and IL-10 in the plasma^90,91^ (Fig. 3), activating ligand downregulation and inhibitory ligand overexpression.^92^ Similar to other immunocytes, NK cells are also inhibited in GBM by upregulation of immune checkpoint molecules and the accumulation of immunosuppressive cells.^93^

T cells play a crucial role in antitumor immunity, especially CD8^+^ T cells. They are present within the TME of GBM. However, their activity is often compromised by a variety of mechanisms,^94^ such as tumor-induced immune checkpoint activation, T cell exhaustion, and infiltration by Tregs.^95^ Additionally, tumor cells release immunosuppressive cytokines, including TGF-β, which inhibit T cell activity.^96^ Midkine, identified as a factor produced by NF1-mutated neurons, can activate T cells, particularly CD8^+^ T cells. They produce CCL4, which consequently in turn acts on microglia, prompting them to release CCL5, which is crucial for the stem cells of low-grade glioma to survive and grow.^97^ This study suggests that CD8^+^ T cells are not always beneficial in killing tumor cells. Based on T cell subtype, their functional status, along with cytokines in TME, infiltrating T cells exhibit protumorigenic and antitumorigenic properties.^98^

Tregs are demonstrated to enhance the progression of GBM via suppressing antitumor immunity and establishing an immunosuppressive environment.^99^ An increase in the number of Tregs in gliomas leads to the production of high levels of IL-10 and TGF-β^91^ (Fig. 3). Moreover, the quantity of Tregs correlates with the World Health Organization grading of brain tumors, and depleting Tregs in mouse glioma models can improve survival rates.^91,100^ Therefore, maintaining a balanced immune function and eliminating Tregs are crucial for preventing the genesis of brain tumors.

B lymphocytes are responsible for the production of antibodies and play a significant role in the adaptive immune response. They can express immunological checkpoints, including PD-L1, that communicate with PD-1 on T cells, resulting in T cell exhaustion as well as the immune evasion of GBM cells.^101^ They can also produce cytokines, including IL-10 and TGF-β, that restrain the function of immunocytes such as T cells and NK cells.^102^ Additionally, B lymphocytes can produce factors that promote angiogenesis, including VEGF, CXCL12, and CXCL13.^103,104^ B cells isolated from GBM patient samples were found to enhance the growth and metastasis of tumor cells by secreting growth factors, including insulin-like growth factor-1 and hepatocyte growth factor. Conversely, Chemokines produced by B cells, such as CXCL13, can influence the TME of GBM, which is associated with enhanced T cell infiltration and improved patient survival.^105^

MDSCs, though scarce in GBM, play a central role in promoting tumor progression, angiogenesis, invasion, and metastasis. They adapt to the TME and suppress T cells via molecules like PD-L1, IL-10, and TGF-β. MDSCs also secrete VEGF for new blood vessel formation, nourishing the tumor, and release matrix metalloproteinases (MMPs) to break down the extracellular matrix, aiding tumor invasion and metastasis.^106^

DCs are pivotal antigen-presenting cells that initiate antitumor immunity by capturing, processing, and presenting tumor antigens to T cells. They engage in bidirectional communication with tumor cells through cytokine and chemokine exchange, which can influence tumor growth and survival. For instance, glioma cells can upregulate thrombospondin-1 (TSP-1) in DCs, impairing their maturation and skewing cytokine secretion toward a Th2 profile, potentially inducing immunosuppression. Leveraging the immunostimulatory properties of DCs, DC-based vaccines, such as DCVax-L, have been developed to enhance the immune response against brain tumors.^107^

### Neuron-immune cell-cancer cell crosstalk

The integration of optic nerve activity in regulating the immune response to cancer growth has been demonstrated in experiments conducted on mice with Nf1 optic gliomas.^108^ In addition to the light-induced, visually experience-dependent neuronal control of the growth of optic gliomas, the Nf1 mutation in neurons also increases the firing of spontaneous action potentials.^108^ This heightened excitability leads to the production of midkine, a heparin-binding growth factor, which acts on T cells to induce the secretion of CCL4.^97^ This, in turn, causes microglia to secrete CCL5, a key mitogen for glioma cells.^109^ A paradigm has been established in which neurons modulate immune cells to facilitate tumor growth; however, this avenue of research is currently underexplored and necessitates further investigation (Fig. 3).

### Neuron-cancer cell crosstalk

Recent findings reveal that normal neurons in the dorsal raphe nucleus, which secrete serotonin, project to the cortex where ependymomas grow. Upon entering ependymoma cells, serotonin binds to histone H3, a protein intricately associated with DNA. Histone serotonylation can modulate tumor growth. In animal models, enhancement of histone serotonylation intensified the growth of ependymomas, whereas its inhibition attenuated tumor progression. This study primarily focuses on the interplay between neuronal signaling, histone modifications, and tumor growth. Although the study does not investigate the involvement of immune cells in the pathogenesis, however they may be indirectly affected.^110^ The interactions between neurons and cancer encompass a plethora of mechanisms.^1,11^ An in-depth exploration of these mechanisms is beyond the scope of the present review, since we focus on the neuro-immune-cancer axis.

## Peripheral nerves regulate the tumor immune microenvironment

### Sensory nerve

Sensory neurons are capable of sensing and modulating immune and inflammatory responses.^111^ Noxious stimuli cause the release of neuropeptides from peripheral nerve fibers, such as CGRP and substance P (SP).^112^ The signaling of SP is mediated via the neurokinin 1 receptor (NK-1R),^113^ and enhances the activity of bone marrow stem cells. SP or NK-1R boosts the survival of activated T cells, triggers macrophages to secrete pro-inflammatory cytokines, and facilitates neutrophil chemotactic and migratory capabilities induced by CCL5.^113–115^ However, in thyroid cancer, SP binding to NK-1R can promote cancer growth, prevent cell death, and increase cancer spread and blood vessel formation.^116^ SP can also promote tumor metastasis by activating Toll-like receptor 7 on breast cancer cells through the action of extracellular RNA.^117^

The CGRP has various effects on components of the immune system. It can exert inhibitory effects, negatively impact innate immune responses, reduce inflammatory tissue injury, protect the host, while also exhibiting pro-inflammatory properties, such as attracting eosinophils to areas of inflammation, enhancing the production of extracellular traps, and facilitating T cell attachment via β integrins regulating.^118,119^ In addition, the neuron excretion of CGRP causes a decrease in levels of cytokine within macrophages as well as DCs, thereby inhibiting antigen presentation to cytotoxic T lymphocytes (CTLs).^120^ After activation of cAMP-dependent protein kinase through downstream receptors, CGRP enhances the secretion of IL-10 and inhibits the nuclear factor κB (NF-κB) activity, afterward, reduces monocytes and the function of macrophages and group 2 innate lymphoid cells recruitment, which are key to both physiological hemostasis and the defense of mucosal barriers. A study has shown that CGRP reduced inflammation in mouse models of LPS-induced pulmonary damage; this mechanism is based on the regulation of macrophage polarization toward M2-type.^120^ CGRP decreased NLRP3 expression in pro-inflammatory M1 macrophages, as well as the expression of proIL-1βmRNA triggered by LPS. Molecules produced by M2 macrophages, such as IL-10, are involved in this skewed polarization after the release of IL-4.^120^ Sensory neurons can also collaborate with immune cells to influence host defense against lung infections and injuries, exacerbate allergic reactions, and their role in lung cancer warrants further investigation.^121^

**Fig. 4:**
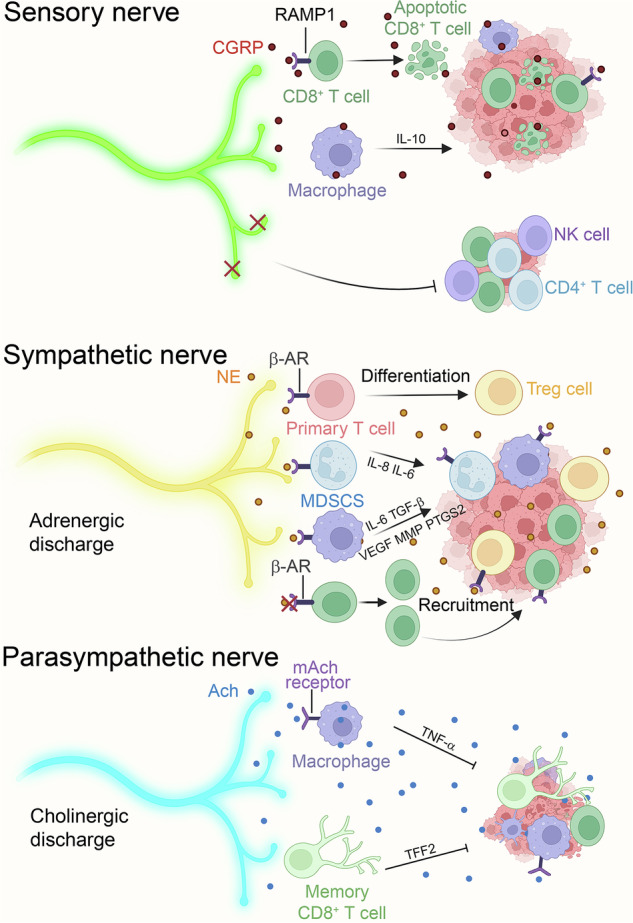
The TIME is regulated by peripheral nerves. Nociceptor neurons interact with melanoma cells, leading to the release of CGRP from nociceptor neurons. CD8^+^ T cells express the CGRP receptor RAMP1. CGRP, by binding to RAMP1, directly increases the exhaustion of cytotoxic CD8^+^ T cells. CGRP significantly enhances the expression of IL-10, a marker of IL-4-induced M2-type macrophages. In a syngeneic oral cancer model using CGRP gene knockout (CGRP KO) mice, a higher infiltration of CD4^+^ and CD8^+^ T lymphocytes and NK cells was observed compared to the control group. NE enhances primary T cells differentiation into Tregs, which prevents effective antitumor immunity and favors further cancer growth and progression. Myeloid cells secrete IL-8 and IL-6 to promote tumor growth. In addition, activation of β-AR can promote cancer growth via secretion of immunosuppressive factors like VEGF, MMP, prostaglandin-endoperoxide synthase 2, IL-6, and TGF-β from macrophages. Blocking β-AR signaling enhances the generation of tumor antigen-specific CD8^+^ T cells in the tumor-draining lymph nodes and increases the frequency of tumor-infiltrating CD8^+^ T cells. Cholinergic activation of mAchR^+^ macrophages and TNF-α secretion inhibits tumor growth. The vagus nerve and TFF2-expressing Tms are suggested to prevent cancer progression. This figure was created with BioRender (https://biorender.com/)

Cranial nerves typically have branches distributed in head and neck cancers and are involved in the regulation of TME. Most of the branches originate from the trigeminal nerve.^122^ CGRP, along with αCGRP isomer, are the main synaptic transmitters in the trigeminal ganglion, utilized by nerves through RAMP1-GPCR interaction signaling.^123,124^ In oral cancer tissue samples, an increase in αCGRP^+^ neuron innervation has been found, alongside a plethora of RAMP1^+^, CGRP receptors, as well as lymphocytes infiltrated in TME.^125^ This suggests that αCGRP plays a significant role in modulating tumor-associated immunity through the RAMP1 signaling pathway, which can be implicated in both innate and adaptive immunity. In a syngeneic oral cancer model utilizing CGRP gene knockout (CGRP KO) mice, researchers observed a marked increase in the infiltration of CD4^+^ and CD8^+^ T lymphocytes, as well as NK cells, compared to wild-type (WT) controls. Furthermore, the CGRP KO group exhibited a notable reduction in tumor size relative to the WT mice, indicating that αCGRP signaling can hinder antitumor immune activity.^126^ Additionally, single-cell RNA sequencing (scRNA-seq) revealed increased expression of RAMP1 in tumor-infiltrating lymphocytes in head and neck squamous cell carcinoma (HNSCC) samples compared to healthy donor palatine tonsil tissues, particularly in CD3^+^ CD4^+^ FOXP3^–^ T cells. Notably, no difference in RAMP1 levels was found between the blood monocytes from HNSCC patients and healthy donors, suggesting the presence of localized, tumor-specific neural modulation of leukocytes.^126^ In mice with melanoma, CGRP^+^ sensory neurons have been found to inhibit antitumoral immune function via the RAMP1 signal, prompting CD8^+^ T cells exhaustion within TIME. Pharmacologic blockade of RAMP1 or knockout of the transient receptor potential vanilloid 1 (TRPV1, a sensory neuron ion channel) caused a reduction of CGRP^+^ signaling in TME, leading to decreased T cell exhaustion, reduced cancer size, as well as extended overall survival rates.^127^ ScRNA-seq of human melanoma tissues illuminated that RAMP1^+^ CD8^+^ T cell exhibited higher levels of exhaustion compared to RAMP1^−^ CD8^+^ T cells, and patients with higher intensity of RAMP1^+^ CD8^+^ T cells had poorer survival rates^127^ (Fig. 4). In the glucose-deprived environment, oral mucosal cancer tissue secreted nerve growth factor (NGF) via activation of c-Jun triggered by reactive oxygen species (ROS). This NGF modulated nociceptive sensory nerves, promoting the production of CGRP. CGRP induced protective autophagy in cancer cells by disrupting the interaction between mTOR and Raptor, mediated by Rap1. The study indicates that nutrient-deprivation therapies targeting glycolysis and angiogenesis may exacerbate the vicious cycle between cancer cells and nociceptive sensory nerves. The use of FDA-approved anti-migraine drugs to block CGRP can significantly enhance the efficacy of tumor starvation therapy.^128^ In another study, the enrichment of CGRP-positive nerves was also found to be associated with poor prognosis in oropharyngeal squamous cell carcinoma(OPSCC), suggesting that CGRP antagonists may improve patients’ quality of life after radiotherapy.^129^ Medullary thyroid cancer (MTC) is a neuroendocrine tumor, it is reported that CGRP is highly expressed in MTC cells, which is linked to poorer disease-free survival. CGRP interacts with its receptor complex, including CALCRL and RAMP1 on DCs, leading to increased Kruppel-Like Factor 2 expression. This interaction disrupts the development of DCs, which are crucial for antitumor immune responses.^130^ In contrast to the aforementioned studies, CGRP can promote the differentiation of Th1 and Tc1 cells while inhibiting Th2 cell differentiation by activating RAMP3. In a mouse model of LCMV Armstrong virus infection, CGRP was shown to be necessary for enhancing antigen-specific Th1 and Tc1 responses. Mice deficient in CGRP (*Calca*^−/−^) exhibited weakened Th1 and Tc1 responses and reduced viral clearance capabilities following infection. This study suggests that the CGRP-RAMP3 axis may enhance T cell-specific tumor-killing functions, but further experiments are needed to confirm this.^131^

### Sympathetic nerve

NE is the most common neurotransmitter released by sympathetic nerves. NE has a significant impact on TIME which is still a matter of controversy.^132^ Myeloid cells, including TAMs, tumor-associated DCs, and MDSCs, are influenced by environmental stress. Cancer cells as well as stromal cells possess adrenergic receptors (ARs), which enhance their resistance against immune cell-mediated killing and apoptosis. β-AR activation promotes the secretion of immunomodulatory cytokines, including IL-8 and IL-6, by cancer cells and myeloid cells.^133–135^ In addition, activation of β-AR can promote cancer growth via secretion of immunosuppressive factors like VEGF, MMP, and prostaglandin-endoperoxide synthase 2, IL-6, and TGF-β from macrophages.^136^ The immunosuppression in TME is further driven by a positive feedback mechanism promoted by GM-CSF secreted by carcinoma, resulting in elevated expression of β2-ARs on MDSCs. Following that, β2-AR signaling inhibits glycolysis as well as promotes lipids oxidation, augmenting the immunosuppressive response of MDSCs that is dependent on fatty acid oxidation and carnitine palmitoyltransferase 1A.^26,137^ Moreover, MDSCs treated with the nonselective β-AR agonist (ISO) exhibit increased expression of ARG1 and PD-L1,^138,139^ contributing to the suppression of T cell-mediated tumor killing, which can be reversed by the use of a β-AR blocker (propranolol). In vitro experiments show that the immunosuppressive effects of MDSCs treated with ISO on the proliferation of CD8^+^ and CD4^+^ T cells are stronger than those of the control MDSCs.^138,139^ Additionally, β-AR activation inhibits type I and II interferon production, which attenuates cell-mediated immune activities against cancer cells as well as oncogenic viruses.^140^

Adrenergic signaling directly influences tumor cells and concurrently facilitates immune evasion. After exposure to NE in pancreatic cancer cells, there is a significant reduction of MHC I and co-stimulatory ligand B7-1, while the increase of immunosuppressive indoleamine-2,3-dioxygenase and B7H-1, which prompts cancer cells to evade recognition by T cells.^141^ In mice with B cell lymphoma, AR activation in hematopoietic cells through ISO leads to cancer growth^142^ which inhibits the proliferation of CD8^+^ T cells, the production of IFN-γ, and the apoptosis of cancer cells, weakens the reaction of CD8^+^ T cells to anti-PD-1 and anti-4-1BB therapy, resulting in a significant reduction in tumor-free survival rates.

To study how adrenergic signaling affects CD8^+^ T cells in cancer, HPV-expressing cancer cells were treated with the STxBE7 vaccine and IFN-α to boost CD8^+^ T cell reactions. Propranolol elevated the CD8^+^ T cell infiltration in TME, but did not enhance their reactivity to tumor cells despite raising activation markers and granzyme B production post-vaccination. However, in tumor-draining lymph nodes, naive CD8^+^ T cells with higher β2-AR expression responded better to β-blockers than the tumor-infiltrating subset, which had reduced β2-AR (Fig. 4). This suggests that using β-blockers early in T cell activation may improve cancer immunotherapy outcomes.^142^

Clinical trials with metastatic melanoma patients have shown that adding a nonselective β-AR blocker to immunotherapy improved survival rates.^143^ Mouse melanoma models suggest that a selective β-AR blocker targeting β2-AR with anti-PD-1 and IL-2 can optimize response and survival rates. Similar benefits were seen in fibrosarcoma and colorectal cancer (CRC) mouse studies with propranolol, which increased T cell infiltration and reduced MDSCs, boosting antitumor immunity, and its effects were amplified when combined with anti-CTLA-4 treatment.^144^

### Parasympathetic nerve

Acetylcholine (Ach) is the most common neurotransmitter released by parasympathetic nerves. Muscarinic ACh receptors (mAchRs) participate in the cholinergic regulation of the immune system.^145^ Activation of mAchRs on T cells elevated the levels of aldehyde dehydrogenase in colon antigen-presenting cells in humans and mice,^145^ which activated Tregs to infiltrate into surrounding tissues. After bilateral subdiaphragmatic vagotomy in mice, the proliferation and invasion of pancreatic ductal adenocarcinoma (PDAC) were promoted, revealing the potential antitumor capability of the vagus nerve.^146^ Tumor growth was inhibited by treatment with bethanechol (a muscarinic agonist and a parasympathomimetic drug) through mAchR-dependent signaling pathways, which was attributed to elevated immune cells infiltration and a large number of CD44^+^ epithelial cells in the pancreas, However, the tumor-inhibiting responses disappeared after bilateral subdiaphragmatic vagotomy.^147^ In the control group, Ach induction of tumor-infiltrating mAchR^+^ macrophages released TNF-α and inhibited tumor growth, while in vagotomized mice, this cholinergic signaling effect was absent^147^ (Fig. 4).

Acetylcholinesterase (AChE) is a degrading enzyme that modulates Ach concentration within the interstitial spaces of tissues. By modulating the activity of AChE, an indirect activation of cholinergic signaling effects can be accomplished. Human pancreatic cancer cells have been shown to exhibit high levels of AChE expression, leading to subsequent investigations on the impact of AChE inhibitors, such as physostigmine and neostigmine, on the pancreatic tumor TME.^148^ Pancreatic cancer models have shown that application of ACh, as well as physostigmine and galantamine, reduced the activity and invasiveness of cancer cells.^101^ That was related to the inactivation of pERK signaling in TME, resulting in a decrease in infiltration of TAMs and secretion of immunosuppressive cytokines.^148,149^ Nevertheless, in a novel genetically engineered, surgically resectable pancreatic cancer mouse model, no correlation has been found between the concurrent use of AChE inhibitors and overall survival rates.^148^ Consistently, no difference in survival rates was observed between patients with high AChE levels and those with low AChE levels in pancreatic cancer. Interestingly, in advanced cancer cells, there is a decreased intensity of choline acetyltransferase (ChAT), the key enzyme for ACh synthesis.^148^

Furthermore, vagal nerve cholinergic signaling is able to promote Trefoil Factor 2 (TFF2) release from memory T cells (Tms) in the spleen, that inhibits inflammation and the development of CRC^150^ (Fig. 4). In a colitis model, TFF2 levels decrease as inflammation resolves, with MDSCs peaking in inflamed tissues. Vagotomy in mice reduced splenic TFF2 expression, while vagal nerve stimulation upregulated TFF2 mRNA. TFF2 knockout mice developed more colon tumors with higher dysplasia and MDSCs infiltration, lacking CD8^+^ T cell infiltration. These MDSCs expressed more PD-L1, inhibited IFN-γ synthesis as well as the CD4^+^ T cell proliferation, and increased expression of IL-17A and IL-1β. CD11b^+^ Gr-1^+^ MDSCs play a pivotal role in the inhibition of CTLs within TME. Vagus nerve and TFF2-expressing Tms are suggested to inhibit cancer development and MDSCs infiltration into TME.^150^ Therapeutic methods proposed include TFF2 overexpression, viral vector transfection, and TFF2-expressing bone marrow transplantation.

The studies highlight the urgent need for deeper investigations into direct downstream effects of cholinergic signaling within TME as well as its interaction with the immune system.

### Neurotransmitters, neuropeptides, neurotrophic factors, and neurometabolites

CNS is rich in neurons that can secrete neurotransmitters and neuropeptides, including NE, ACh, γ-aminobutyric acid (GABA), serotonin (5-HT), dopamine (DA), and histamine (HA). Found beyond the CNS, these same neurotransmitters and neuropeptides are also detected in peripheral tissues. CGRP, NE, and ACh have been discussed previously and will not be reiterated. In peripheral tissues, GABA, 5-HT, DA, and HA are often secreted by immune cells or neuroendocrine cells, and receptors for them exist on immune cells.^151,152^

B lymphocytes synthesize and secrete the neurotransmitter GABA, which promotes the differentiation of monocytes into anti-inflammatory macrophages. These macrophages secrete IL-10 and inhibit the cytotoxic function of CD8^+^ T cells.^151^ Additionally, researchers have found that GABA can bind to GABAA receptors on CD8^+^ T lymphocytes, suppressing antitumor immunity and promoting tumor growth.^151^ In non-small cell lung cancer (NSCLC) and CRC, aberrantly expressed glutamate decarboxylase 1 reprograms glutamine metabolism for GABA synthesis. A study of patient specimens shows that elevated GABA levels correlate with a low survival rate. GABA activates GABAB receptors, inhibiting the activity of glycogen synthase kinase 3β, which leads to the enhancement of the β-catenin signaling pathway. This GABA-mediated activation of β-catenin not only promotes tumor cell proliferation but also suppresses the infiltration of CD8^+^ T cells in the tumor.^152^

5-HT is commonly secreted by platelets. An interesting phenomenon shows that fluoxetine, a selective 5-HT reuptake inhibitor, has slowed the growth of melanoma by modulating the immune system. It has been found that fluoxetine inhibited the progression of melanoma by inducing T cells to proliferate through mitogen signals. In pancreatic and gastric cancer models, 5-HT has been shown to upregulate the expression of PD-L1 through histone serotonylation and subsequent epigenetic regulation of immune checkpoint expression.^153^ Additionally, the precursor of 5-HT, tryptophan, is metabolized by indoleamine-2,3-dioxygenase (IDO) and tryptophan-2,3-dioxygenase (TDO) into kynurenine, which is involved in neuroactivity and immune modulation and is associated with neurodegenerative diseases and cancer.^153^

Recent studies highlight the link between the DA system and cancer. DA influences tumor behavior via its receptors, categorized as D1-like (DRD1, DRD5) and D2-like (DRD2, DRD3, DRD4), which vary in tumor expression and function. DRD3 expression is linked to liver cancer prognosis; lower levels correlate with worse survival outcomes. DRD3 agonists reduce HCC cell proliferation and invasion, while antagonists have the reverse effect.^154^ DA via DRD5 promotes CD8^+^ T cell differentiation into CD103^+^ tissue-resident Tms, enhancing the antitumor immune response. In CRC, higher DA levels are linked to better patient survival and increased CD8^+^ T cell infiltration.^155^ DA transporter antagonists like vanoxerine are being explored for cancer treatment, vanoxerine inhibits colorectal cancer stem cells (CSCs) function by downregulation of G9a histone methyltransferase and activates endogenous transposable elements and type I interferon responses to enhance tumor lymphocytic infiltration, offering a new strategy to boost the antitumor immune response.^156^ In the co-culture system of glioma stem-like cells (GSCs) spheres with brain organoids, the DRD1 inhibitor, SKF83566, suppressed GBM stemness and invasion through the DRD1-c-Myc-UHRF1 interaction. The ability that SKF83566 can cross the blood–brain barrier makes it a promising small-molecule drug for GBM.^157^

Elevated HA levels are found in cancer patients’ blood and tumors, likely due to increased L-histidine decarboxylase (HDC) in cancer cells. The role of HA and its receptors in tumors is debated, but HA is known to influence the TIME.^158^ Cimetidine, an H2 antagonist, inhibits lung tumor growth in mice by reducing CD11b^+^ Gr-1^+^ MDSCs.^159^ Increased HA and H1 receptors in the TIME can promote an immunosuppressive M2-like macrophage phenotype, which regulates the immune checkpoint VISTA to inhibit T cells. H1 antagonists can counteract this, enhancing responses to PD-1 monotherapy in patients on antiallergic drugs.^160^ The H4 receptor, expressed in immune cells including T cells, is a potential target for treating inflammation and autoimmune diseases. Activation of the H4 receptor can influence T cell function, either directly or indirectly, by affecting other immune cell subsets. In a breast cancer model, the absence of the H4 receptor correlated with slowed tumor growth and improved survival rates.^161^

In HNSCC, GDNF, a neurotrophic factor secreted by nerves, can enhance the expression of PD-L1 on tumor cells by activating the JAK2-STAT1 signaling pathway. The interaction between PD-L1 and PD-1 on T cells aids tumor cells in evading immune surveillance and attack.^162^

A recent study has discovered that CNS-enriched metabolite N-acetylaspartate is highly expressed in breast cancer cells. It disrupts the formation of the immunological synapse by promoting the P300/CBP-associated factor (PCAF)-induced acetylation of laminA-K542, thereby inhibiting the cytotoxicity of NK cells and CD8^+^ T cells, ultimately impairing antitumor immunity.^163^

Neurotransmitters, neuropeptides, neurotrophic factors, and neurometabolites can exert diverse influences on tumorigenesis and tumor progression. Investigating their specific roles in various cancers could facilitate the modulation of these pathways to complement antitumor therapies. Through elucidating the mechanisms by which each neurotransmitter interacts within the TME, thereby influencing cancer cell behavior, precision therapies could be engineered to optimize these interactions, potentially augmenting the efficacy of oncology treatment strategies. This strategy may offer enhanced refinement and individualized approaches to cancer therapy, leveraging a triangular relationship between neurons, immune cells, and cancer cells.

### Glial cells

Within the oncological milieu, glial cells constitute a significant component of the neuro-immune axis. Within the peripheral nervous system, the myelin sheath, predominantly formed by Schwann cells, plays a crucial role in the reparative processes of axonal integrity.^164^ Furthermore, these Schwann cells augment the chemotactic migration of immune cells through the secretion of chemotactic cytokines. Activated glial fibrillary acidic protein (GFAP^+^) Schwann cells, when co-cultured with melanoma-conditioned medium, upregulated expression of genes participating in immune surveillance and chemotaxis, such as IL-6, TGF-β, and VEGF.^165^ It has been shown that CCL2, synthesized and secreted by Schwann cells, promoted proliferation in co-culture experiments with HeLa and ME180 cervical cancer cells.^166^ The modulation of the TIME by CCL2 is associated with immune suppression, which is related to poor overall survival rates in human melanoma.^167^ After the secretion of CCL2 into the TME, TAMs polarize into the M2-type, promoting tumorigenesis and suppressing antitumor immune responses.^167^ In addition, Schwann cells also modulate the recruitment of suppressive MDSCs and enhance the growth of melanoma, pancreatic, and prostate cancers.^167–169^

Furthermore, previous studies have indicated that Schwann cells possess Toll-like receptors (TLRs) and can recognize damage-associated molecular patterns (DAMPs), especially in diseases involving nerve damage.^169,170^ DAMPs are produced by various cancers, including pancreatic cancer, breast cancer, CRC, and GBM, that are detected by TLRs on macrophages and DCs within TME.^171^ It is proposed that TLRs expressed on Schwann cells may play a role in the immune response within the TME by recognizing DAMPs secreted by the tumor. Upon TLR activation, innate immune responses are promoted, including the maturation of antigen-presenting cells and subsequent T cell activation.^172^ Additionally, Schwann cells express CD74, CD1a, CD1b, CD1d, B7-1, BB-1 and cell adhesion molecule CD58, supporting their role as antigen-presenting cells. Further investigations are needed to elucidate the function of these molecules within the TME.^173–175^

### The interaction between peripheral neuroendocrine cells and immune cells

Neuroendocrine cells originate from the neural crest cells of the neural ectoderm and the endoderm cells encoded by the neuroepithelium. They are epithelial cells with many characteristics of neuronal cells, including numerous secretory vesicles and the ability to sense environmental stimuli.^176^ Upon sensing danger, neuroendocrine cells can communicate with nerve terminals, triggering neural reflexes that protect the body from harm.^177^ Peripheral neuroendocrine cells are distributed throughout various systems in the body, such as the respiratory tract, gastrointestinal tract, prostate, pancreas, and so on.^119,178,179^

Neuroendocrine cells can interact with immune cells to regulate inflammatory responses within the body. For instance, neuroendocrine cells in the respiratory tract can interact with eosinophils through CGRP and extracellular traps, exacerbating asthma.^119^ In the gut, IL-33 can be sensed by enterochromaffin cells, leading to the release of 5-HT. This release, upon activation of intestinal neurons, promotes intestinal motility. Mechanistically, IL-33 triggers an influx of calcium ions through an atypical signaling pathway, inducing the secretion of 5-HT, which emphasizes the significant role of the IL-33-ST2 signaling pathway in regulating intestinal motility and host defense. The findings reveal an immuno-neuroendocrine axis that rapidly modulates the release of 5-HT to maintain intestinal homeostasis.^178^

Unregulated proliferation of neuroendocrine cells can lead to the development of neuroendocrine cancers, such as small cell lung cancer (SCLC)^180^ and gastrointestinal neuroendocrine cancers (GEP-NENs).^181^ It has been found that SCLC tumors possess an immunosuppressive landscape, compared to normal adjacent tissues (NATs), tumor tissues exhibit greater lymphocytic infiltration and less myeloid cell infiltration. This suggests that adaptive immunity plays a more significant role in TME, while innate immune responses are more crucial in normal lung tissue. T cells from both NATs and TME are primarily composed of CD8^+^ T cells and express high levels of cytotoxicity markers such as GZMA/B/H/K, PRF1, NKG7, IFNG, GNLY, and CXCL13, indicating significant immune surveillance in SCLC.^180^ Compared to cytotoxic T cells from NATs alone, T cells from the TME display increased diversity, including four states of heterogeneous activation based on naive, cytotoxic, exhausted, and proliferative characteristics. Detailed classification of T cells in SCLC also reveals the expression patterns of dysfunction and exhaustion markers (such as PD-1, CTLA-4, TIM3, LAG3, TIGIT, and LAYN), which may serve as targets for immunotherapy.^180^ In the field of digestive system neuroendocrine tumors, different regions of GEP-NENs exhibit distinct TIME characteristics. For instance, pancreatic neuroendocrine tumors (Pan-NENs) differ from small intestine neuroendocrine tumors (SINENs) in terms of T cell infiltration and PD-L1 expression.^182^ Studies have shown that both tumor and non-tumor areas in Pan-NENs have a high level of T cell infiltration, including CD3^+^, CD45RO^+^ (Tms), and CD8^+^ T cells. Compared to SINENs, Pan-NENs exhibit more T cell subset infiltration in non-tumor areas, suggesting a more active immune response in Pan-NENs. PD-L1 expression is quite common in Pan-NENs, approximately 97%, which may be related to the tumor’s immune evasion mechanisms. In contrast, PD-L1 expression in SINENs is not widespread, which could affect the efficacy of immunotherapy and the TIME.^182^

The presence of neuroendocrine differentiation in tumors may indicate a poor prognosis. For instance, in prostate cancer, patients with neuroendocrine cell positive staining assessed by objective criteria have been shown to have worse outcomes in terms of biochemical progression and prostate cancer-specific survival, even among patients with low-risk cancer.^179^ This finding suggests that future studies should establish the precise quantitative thresholds for reporting neuroendocrine cell staining to more accurately identify patients at higher risk of progression. This could help improve clinical management strategies.^179^

## The impact of circadian rhythms on tumor immunity

**Fig. 5:**
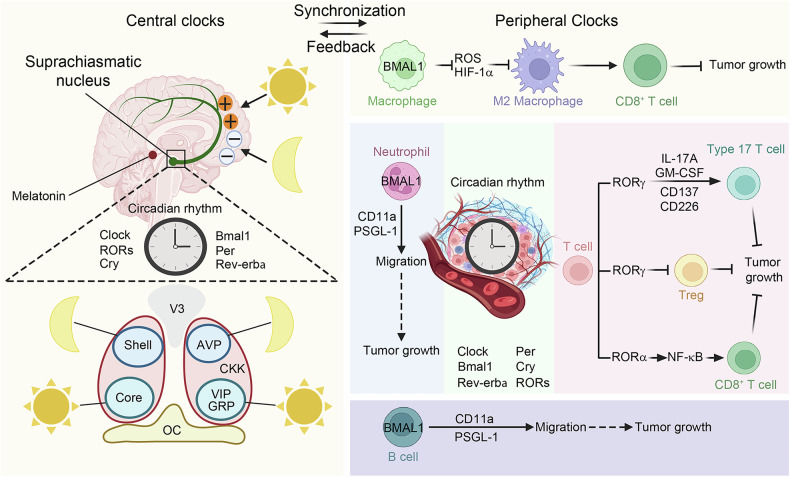
The impact of circadian rhythms on tumor immunity. The circadian clock system is composed of central and peripheral clocks. The central clock, located in the SCN of the anterior hypothalamus, operates autonomously and coordinates peripheral clocks throughout the body by sending signals. SCN, which are located immediately above the optic chiasm in the anterior-ventral area of the hypothalamus, are believed to include the pacemaker of this clock. This circadian clock reset comes about through the retinohypothalamic tract, which sends light information directly from the retina to a subset of SCN neurons. A pineal hormone known as melatonin is most abundant in the blood at night and least prevalent during the day. Its secretion is governed by a rhythm-generating process in the SCN, which is regulated by light. Melatonin is not only regulated by the circadian oscillator but also provides the oscillator with a feedback signal for darkness. Bilateral structure of the SCN and its “core” and “shell” subregions with VIP and gastrin-releasing peptide (GRP) in the light-responsive core and arginine vasopressin (AVP)-expressing cells in the shell, the diurnal expression of VIP in the suprachiasmatic nucleus is influenced by the light/dark cycle, and VIP is involved in the regulation of circadian rhythms and immune responses. The CCK neurons exhibit photic antagonism and can form a negative feedback loop with VIP neurons, inhibiting the activity of VIP neurons. At the molecular level, the circadian rhythms from both the central and peripheral clocks are similar. BMAL1 in macrophages inhibits the production of HIF1α and reactive oxygen species (ROS), inhibiting the polarization of macrophages to the M2-type, which enhances the activation of CD8^+^ T cells and suppresses tumor growth. RORγ enhances the differentiation and effector functions of Th17 cells. RORγ reduces the levels of Tregs. The activation of RORα can enhance the activity and function of CD8^+^ T cells by alleviating the NF-κB pathway. BMAL1 in B cells and neutrophils can change their migration ability via modulating the expression of promigratory molecules that may affect tumor growth. optic chiasm (OC); the 3rd cerebral ventricle (V3). This figure was created with BioRender (https://biorender.com/)

The circadian rhythm is an internal timekeeping mechanism controlled by the neural system within living organisms that regulates many physiological and behavioral responses, including the sleep-wake, feeding-fasting, and activity-rest cycles. The circadian clock system comprises central and peripheral clocks.^183^ The central clock, located in SCN, operates autonomously and coordinates peripheral clocks throughout the body by sending signals.^184^ In rats, the diurnal expression of vasoactive intestinal peptide (VIP) in SCN is influenced by the light/dark cycle, and VIP plays a significant role in the regulation of circadian rhythms and immune responses.^185^ Furthermore, the cholecystokinin (CCK) neurons in the SCN are instrumental in maintaining the robustness of the circadian rhythm. Notably, across diverse photoperiods, a surge in the calcium activity of CCK neurons is observed just before the commencement of mice’s nocturnal activity, suggesting their role in heralding the onset of nighttime behavior. These CCK neurons engage in a negative feedback mechanism with VIP neurons, effectively dampening the activity of the latter. This interaction is crucial as it mitigates the desynchronizing effects of light exposure on the SCN, thus preserving the stability of rhythmic behavior, particularly during extended photoperiods.^186^ The SCN regulates the diurnal fluctuations of immune cell traffic in peripheral lymph nodes through the autonomic nervous system.^185,187^ Cortisol and adrenaline can control the opposite diurnal rhythms of T cell subsets, with these hormones modulating immune responses through various signaling pathways.^188^ Adrenergic innervation governs the diurnal recruitment of leukocytes into tissues.^189^ Sleep deprivation or irregular sleep patterns may lead to a decline in immune function, thereby weakening the body’s immune surveillance and clearance capabilities against tumors.^190^ On the other hand, physiological activities and psychological stress during the waking state may affect the function of immune cells through neuroendocrine pathways, thereby affecting tumor immunity^191^ (Fig. 5).

At the molecular level, the circadian rhythms from both the central and peripheral clocks are similar and are controlled by clock genes. Clock genes are a class of genes within organisms that regulate circadian rhythms.^185^ The proteins encoded by these genes form intricate regulatory networks to sustain biological rhythms. The central molecular clock mechanism is regulated by transcription-translation feedback loops (TTFL).^192–194^ Brain and muscle ARNT-like protein 1 (BMAL1) and Circadian locomotor output cycles kaput (CLOCK) form part of positive feedback loop, which bind to E-box motifs as well as elevate transcriptional repressors, such as the cryptochrome (CRY1 and CRY2) and period (PER1, PER2, and PER3) genes. CRY and PER form a complex that enters the nucleus and inhibits the CLOCK-BMAL1 complex.^195–197^ Additionally, the CLOCK-BMAL1 complex modulates the level of nuclear receptors REV-ERBα/β^198^ as well as Retinoid X receptor-related orphan receptors (RORs), which inhibit or activate BMAL1, forming a second feedback loop.^191–193,199,200^ SCN neurons maintain synchronization through synaptic connections, couple with each other, and transmit signals to peripheral clocks via neural and neuroendocrine stimulation, achieving a coherent rhythm throughout the organism.^185^

### Circadian disruption and cancer

Circadian disruption is implicated in the pathogenesis of various diseases, including tumor.^201–203^ There is a growing body of evidence suggesting that circadian disruptions, such as night shift work and eating late at night after 9:30 PM, known as “night eaters,” increase the risk of cancer^204–207^ and are related to heightened tumor metastasis in several cancer types, such as breast cancer,^208–210^ NSCLC,^211^ HCC^212^ and CRC.^213^ The significance of this direction is also supported by studies in mouse models, which demonstrate that the circadian clock plays a crucial role in tumorigenesis and in modulating the efficacy of radiotherapy^214,215^ and chemotherapy.^216,217^ Mechanistically, the circadian clock can influence cancer development via regulating various hallmarks of cancer,^218,219^ including DNA damage, apoptosis, cell cycle and senescence,^192,204,220^ proliferation,^192,210^ metabolism,^191,219,221^ replicative immortality,^222^ and genomic instability and mutation.^223^

CSCs are a subpopulation of cancer cells with self-renewal capabilities that significantly promote tumor initiation, metastasis, and therapy resistance.^224^ Elevating proofs suggest that the circadian clock is crucial for sustaining the stemness of CSCs across diverse sorts of cancer, such as acute myeloid leukemia (AML) and GBM.^225–227^ Particularly, in AML, pharmacological along with genetic disruption of circadian clock mechanism leads to CSC differentiation,^227^ Furthermore, within GBM, it impairs the stemness of GSCs and leads to GSC cell cycle arrest and apoptosis.^225,226^ The compound SR8278, which can reduce PER2 expression, may protect mice from pituitary adenoma development by restricting cell cycle progression.^228^ These findings indicate that core components of the circadian clock modulate key biological characteristics of cancer cells across cancer types.

### Circadian disruption in the TIME

In addition to the aforementioned effects on cancer cells, circadian disruption also affects TIME.^203^ In vivo findings from mouse models of breast cancer and melanoma indicate that circadian disruption notably promotes cancer progression, while also inducing a shift toward an anti-inflammatory macrophage phenotype through raising the ratio of TAMs as well as Tregs, and reducing the infiltration and activity of CD8^+^ T cells.^229,230^ Similarly, bioinformatics analyses of gene expression data from human cancer tissues have uncovered correlations between clock genes and infiltration of immunocytes and proliferation of cancer cells.^179^

Clock genes in tumor cells can influence tumorigenesis by modulating the TIME. For instance, in GBM, high levels of CLOCK in GSCs are associated with an increase in microglia in TME, mediated by the transcriptional regulation of chemokine-like protein 3.^225^ The deficiency of Bmal1 in melanoma cells also impacts immunocytes within TME, including CD8^+^ T cells and TAMs.^231^ In kidney clear cell carcinoma (KIRC) and breast cancer, the expression of clock genes (such as CLOCK, BMAL1, and PER3) in cancer cells exhibits rhythmic fluctuations. That is also related to infiltration of macrophages, neutrophils, as well as DCs.^220,232^ Multi-omics analyses of KIRC and NSCLC show that clock genes (such as CLOCK, BMAL1, CRY1, CRY2, PER1, PER2, PER3, RORA, and REV-ERBα/β) are associated with infiltration of lymphocytes, including B cells, CD8^+^ and CD4^+^ T cells.^220,233^ Similarly, patient-derived GSCs exhibit diurnal oscillations independent of tumor genetics,^226^ and the expression of CLOCK in GBM is correlated with reduced levels of activated CD8^+^ T cells.^225^ Furthermore, genomic mutation analysis show that clock genes often mutate in cancer patients, which in turn can induce genomic instability, correlate with the exhaustion of T cells (such as CD8^+^ and CD4^+^ T cells), and are associated with the upregulation of immune suppressive molecules, including PD-L1 and CTLA-4.^234,235^ In vivo outcomes from both syngeneic or allogeneic leukemia models have shown that the recruitment and implantation of leukemia cells/leukocytes was modulated by circadian clock. While the disruption of the circadian rhythm increased the tumor burden.^236^ In T cell acute lymphoblastic leukemia, key components of the circadian clock, CLOCK, along with BMAL1, can influence the activity of leukemia-initiating cells through regulating the JAK/STAT axis.^200^

Clock genes in immune cells can also influence tumorigenesis. When TAMs polarized toward an immunostimulatory phenotype, the expression of BMAL1 in macrophages was elevated. Knockout of BMAL1 in macrophages resulted in the upregulation of HIF1α and accumulation of reactive oxygen species (ROS), as well as a decrease of nuclear factor erythroid 2-related factor 2, thereby modulating the synthesis of pro-inflammatory cytokines.^237,238^ Consequently, compared to Bmal1 WT mice, mice with bone marrow-specific Bmal1 knockout exhibited increased tumor growth and alternatively activated TAMs.^237^ Similarly, co-injection of Bmal1 knockout macrophages with cancer cells enhanced cancer growth as well as inhibited CD8^+^ T lymphocytes infiltration versus WT macrophages.^237^ Therefore, these emerging evidences emphasize that BMAL1 in macrophages is crucial for inhibiting tumor progression and promoting antitumor immunity (Fig. 5). The activation of REV-ERBα can inhibit CCL2 and its downstream signaling (such as ERK and p38 MAPK), thereby suppressing the adhesion and migration of macrophages.^239^ Transcription factor, RORγt, is an intracellular clock component that controls the differentiation of CD4^+^ Th17 cells, which produce IL-17, depending on the circadian clock.^240^ Activation of RORγ with synthetic agonists can enhance the differentiation and effector functions of Th17 cells and reduce Tregs through modulating cytokines/chemokines, co-stimulatory receptors, as well as immune checkpoint molecules.^241^ Thus, co-culture and co-injection of CD8^+^ Tc17 cells and EG7 lymphoma cells treated with RORγ agonists increased apoptosis in vitro and inhibited tumor growth in vivo.^241^ Furthermore, studies in mouse models of breast cancer and CRC showed that RORγ signaling prevented cancer development as well as prolonged the survival of the animal hosts by promoting antitumor immune responses.^241^ It is noteworthy that the impact of RORγ activation on Tregs may not be attributed to its circadian clock, as Tregs lack intrinsic circadian oscillators.^242^ Besides RORγ, the activation of RORα can enhance the activity and function of CD8^+^ T cells via alleviating the NF-κB pathway as well as maintaining the balance of cholesterol metabolism in CD8^+^ T cells. Eventually, these activated CD8^+^ T cells induce apoptosis in CRC cells^243^ (Fig. 5). In conclusion, the studies demonstrate that specifically targeting RORs of T cells will be a potential approach for immunotherapy. The particular deletion of Bmal1 in B cells, neutrophils, or endothelial cells in mice can eliminate the diurnal variation in the expression of promigratory molecules, including integrin αL (CD11a), P-selectin glycoprotein ligand 1 (PSGL-1), intercellular adhesion molecule 1, or vascular cell adhesion molecule 1, thereby eliminating the rhythmic homing of B cells and neutrophils^185^ (Fig. 5). DCs exhibit a rhythmic migration to the tumor-draining lymph nodes in a manner that is dependent on the diurnal expression of the co-stimulatory molecule CD80, which in turn governs the circadian rhythmic response of tumor antigen-specific CD8^+^ T cells.^244^ However, the researchers did not further investigate the correlation between the circadian expression of CD80 and clock genes, which warrants further investigation.

The overexpression of CRY1 in endothelial cells can inhibit the activation of pro-inflammatory cytokines, such as IL-1β, IL-6, and TNF-α, adhesion molecules (such as VCAM-1, ICAM-1, and E-selectin), and the NF-κB pathway, all of which impair the adhesion of monocytes.^245,246^

In addition to the previously discussed cell types in the TME, the complement system must also be considered. It is closely related to the circadian clock^247^ and plays a key role in enhancing tumor progression by promoting MDSCs infiltration and restricting CD8^+^ T cell-mediated immune responses.^248^

Although an elevating number of studies focus on the role of clock genes in TME, there is a paucity of research on how the CNS and central clock regulate peripheral clocks. Additionally, there is a lack of research on how alterations of clock genes in the TME impact central rhythms. Further studies are warranted to elucidate the complete circuitry between rhythms within TME and central rhythms.

### T cell-targeted immunotherapies and the role of circadian regulation

Current immunotherapies targeting T cells include immune checkpoint inhibitors (ICIs) and CAR-T cell therapy.^249^ Considering the significant role of the circadian clock in modulating interactions between cancer cells and TME to influence T cell function, as previously highlighted, a deeper comprehension of this interplay could potentially result in approaches that enhance the efficiency of immunotherapy.^250^ Preclinical studies in mice have shown that the use of RORγ agonists can promote the antitumor immunity of CAR-expressing Th17 cells, providing long-term protection against tumors.^251^ Furthermore, the crosstalk between cancer cells and the TME, regulated by circadian clock components, is associated with an increased infiltration of MDSCs, which consequently impairs T lymphocyte functions and upregulates immune checkpoint molecules.^220,225,232,233,235,252^ These studies indicate that targeting the TME-cancer cell crosstalk regulated by circadian clock components may enhance the antitumor efficiency of ICIs. In fact, this concept is supported by clinical proof, where the antitumor effects of nivolumab (an anti-PD-1 antibody) in patients with advanced pulmonary cancer were notably higher in the morning treatment group compared to the afternoon treatment group.^253^ Since CD8^+^ T cells exhibit rhythmic oscillations within TME, adjusting the timing of CAR-T cell therapy or ICI treatments according to this rhythm could improve efficacy.^246,254–257^ Similarly, scheduling radiotherapy and chemotherapy according to a patient’s circadian rhythm may enhance treatment effectiveness and reduce side effects.^258^ Additionally, a clinical trial testing the combination of RORγ agonists with anti-PD-1 antibodies for patients with metastatic NSCLC is underway (NCT03396497).

These findings indicate that the circadian clock may influence the clinical response to ICI treatment and CAR-T cell therapy. Further investigations focused on understanding the interactions between cancer cells and the immune system, as regulated by circadian clock components, will aid in the design and development of novel, effective, circadian clock-oriented immunotherapies.

## The impact of stress on tumor immunity

**Fig. 6:**
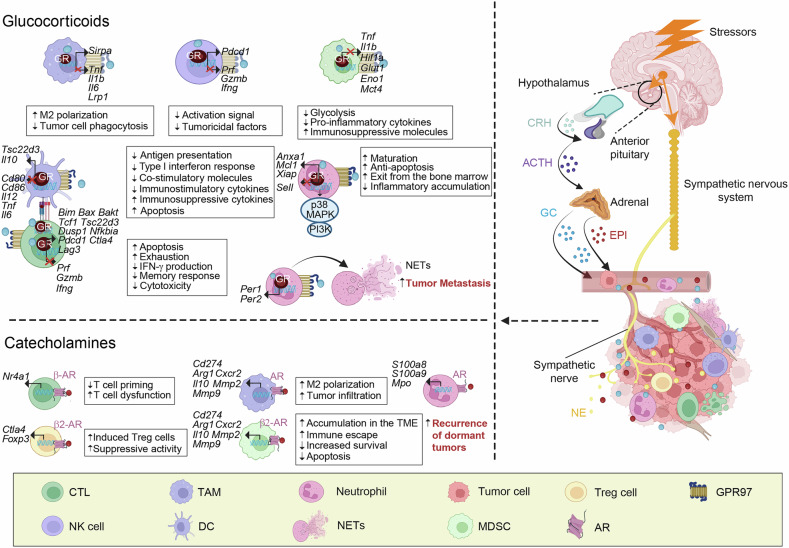
The impact of stress on tumor immunity. Stress activates the HPA axis and sympathetic nerves, resulting in elevated GCs and CAs in the blood and TME, which can engage GR, GPR97, and CA receptors on immune cells. This leads to transcriptional reprogramming, modified signal transduction, and functional alterations. TAM-mediated clearance of tumor cells is disturbed by repression of *Lrp1* and the elevation of *Sirpa*. GCs can also drive TAMs toward an M2-like phenotype and inhibit the expression of genes *Tnf*, *Il1b*, and *Il-6*. GCs dampen the cytolytic activity of NK cells by reducing histone acetylation of genes *Prf*, *Gzmb*, and *Ifng*. They also limit the production of IFN-γ by upregulating the gene *Pdcd1*. GR signaling enhances MDSC immunosuppression by repressing *Hif1α*, *Glut1*, *Eno1*, *Mct4*, *Tnf*, and *Il1b*. GCs confer immunosuppressive properties on DC by downregulating genes *Cd80*, *Cd86*, *Il-12*, *Il-6*, and *Tnf*, while augmenting the expression of genes *Il-10*. The transcription of *Tsc22d3* is elevated in DC, which impairs type I interferon responses and MHC class I/II antigen presentation pathways, thus compromising antitumor immunity. GC-induced downregulation of anti-apoptotic molecules leads to apoptosis of DCs. By stimulating the expression of *Bim*, *Bak1*, and *Bax* in T cells, GCs can activate the mitochondrial pathway of T cell apoptosis. They also dampen the function of T cells by upregulating genes *Tsc22d3*, *Dusp1*, *Nfkbia*, *Pdcd1*, *Ctla-4*, and *Lag3*, while downregulating *Prf*, *Gzmb*, and *Ifng*. They can drive various transcriptomic and epigenetic changes that favor the differentiation of Tregs, whereas strongly restraining memory precursor T cells, CTLs, and Th1 cells differentiation via upregulation of *Tcf1*. GCs block the accumulation of neutrophils in inflamed tissues by downregulating *Sell* and upregulating *Anxa1*. Moreover, GCs exhibit an anti-apoptotic effect on neutrophils by directly activating p38 MAPK and PI3K and upregulating genes *Mcl1* and *Xiap*. They control the expression of key clock genes (*Per1* and *Per2*), leading to an abnormal “aged” phenotype in neutrophils and the formation of NETs, which promote the migration of cancer. Activation of β-ARs in CTLs leads to upregulation of *Nr4a1*, resulting in T cell dysfunction. CAs also promote tumor infiltration and M2 polarization of TAMs, thus accelerating the progression and metastasis of cancer. Upon stimulation with NE, neutrophils rapidly release S100A8/A9 and activate myeloperoxidase to generate oxidized lipids, which further reactivate dormant tumor cells. β2-AR activation can facilitate the conversion of naive T cells to Treg cells and enhance their suppressive activities by increasing CTLA-4 expression. β2-AR signaling in MDSCs leads to downregulation of apoptosis-related genes and upregulation of *Arg1* and *Cd274*. The β2-AR axis also promotes tumor progression via the CXCL5–CXCR2 axis and MMPs, which enhance the mobilization of MDSCs and local immunosuppression. This figure was created with BioRender (https://biorender.com/)

Stress is the physical and emotional response experienced by individuals when encountering life’s challenges and is governed by the nervous system. Chronic stress activates the sympathetic nervous system and the HPA axis, resulting in elevated release of catecholamines (CAs) and GCs.^259,260^ These are referred to as stress-associated immunomodulatory molecules which exhibit immunosuppressive or immunostimulatory functions based on the cells targeted, stressors intensity, sustained period, as well as interaction with immune reaction.^261–263^ Despite the widespread expression of GC receptors (GRs), the immunomodulatory effects of GCs are highly cell type-specific^264,265^ depend on different transcriptional outputs and post-translational modifications (PTMs) of the receptors^266^ (Fig. 6). Dopamine metabolism, transmission, conversion, neuronal innervation, and release are all affected by stress,^267–269^ that promotes primary CD8^+^ T cells homing,^270^ restricts NK cells and the production of IFN-γ,^271^ and mitigates the activation of the NLRP3 inflammasome^272^ through interacting with different dopamine receptors. A surge in NE during acute stress can rapidly enhance the antigen capture of DCs through α2-AR-triggered PI3K and ERK1/2 signaling pathways.^273^ However, stimulating β2-AR on DCs results in the priority release of IL-23, thereby enhancing Th17 response and reducing the Th1 response in CD4^+^ T cells.^274^ β2-AR signaling activation by NE is able to magnify the inhibitory capacity of Tregs through the overexpression of CTLA-4.^275^ Chemogenetic activation of the sympathetic nervous system or administration of AR agonists can imitate stress states within murine models that cause vascular constriction, lessen blood supply locally, as well as trigger rapid oxygen deprivation, impairing T cell movement and defense mechanisms.^44^ The above studies suggest that the characterization and density of the receptors specific to diverse immunocytes determine neuroendocrine modulation of immune responses.^276,277^ This intricate interplay between the stress response and the immune system highlights the complex regulatory network that can influence immune function and has implications for understanding the role of stress in immune-related diseases, including cancer.

Stress has been shown to regulate tumor immune surveillance, with increased UV-induced squamous cell carcinoma sensitivity in restrained mice linked to elevated Tregs and reduced IFN-γ production by T cells.^278^ High stress levels also correlate with impaired NK cell function in ovarian and breast cancer patients.^22^ Understanding the TME’s immune dynamics and their stress-induced molecule alterations is vital for enhancing immunotherapy effectiveness. The neuroendocrine-immune system interplay significantly shapes the antitumor immune response, offering new therapeutic avenues.

### GCs

Synthetic GCs exhibit strong anti-inflammatory and immunosuppressive effects at pharmacological doses. However, stress-induced elevations in endogenous GCs can either suppress or enhance immune responses, depending on factors such as dosage, timing, duration, target cells, and associated transcriptional changes.^279^ During acute stress, the PVN uses GC signaling to control leukocyte migration, while exercise circuits mobilize neutrophils to injury sites, indicating brain regulation of leukocyte distribution and function.^280^ In rodents with tumors, stress from surgery or environmental factors can reduce the effectiveness of immunotherapy, while blocking GC and ARs can enhance NK cell activity and survival.^281^ Repeated social defeat stress in mice impairs the efficacy of chemotherapy, cancer vaccines, and ICIs.^282^ This stress preconditioning elevates GCs, reducing NE and 5-HT levels, and impairs DC function,^283,284^ inducing T cell impairment along with therapeutic inefficacy.^282^ The efficiency of chemotherapeutic interventions or vaccines can be negated by clinical doses of synthetic GCs or overexpression of TSC22 domain family member 3 (*Tsc22d3*) in DCs of unstressed mice, but using a GC receptor antagonist or deleting *Tsc22d3* can counteract stress-induced immunosuppression.^282^ Mifepristone has shown benefits in reducing tumor progression and improving life quality for patients suffering from various tumor types.^285–287^ GCs inhibit neutrophil aggregation within areas of inflammation by decreasing Sell and increasing Anxa1. In addition, they can activate p38 MAPK and PI3K, increase *Mcl1* and *Xiap* gene expression, as well as block neutrophil apoptosis.^288–290^ In a breast cancer model, GCs alter neutrophil circadian rhythms, promoting NET formation and tumor metastasis^291^ (Fig. 6). Emotional stress in patients with advanced NSCLC treated with ICIs is associated with poorer outcomes, suggesting the importance of addressing emotional stress in cancer management, as it can activate the HPA axis and increase GCs release, potentially affecting ICI effectiveness.^292,293^ The concept of “psycho-biomarker” has been proposed for potential application in various cancers.

GCs are produced by monocyte-macrophage lineage cells in TIME as well. These locally produced GCs can bind to GR and upregulate the transcription factor TCF1, leading to the dysfunction of CD8^+^ T cells. This in situ GC secretion is related to the failure of immunotherapy in preclinical models of melanoma and in patients with melanoma.^261^ In NSCLC patients, scRNA-seq has indicated an association between the expression of TSC22D3 in tumor-infiltrating DCs and peripheral blood monocytes. Additionally, the studies revealed a positive association between circulating levels of cortisol and the levels of TSC22D3 in monocytes of the peripheral blood and the emotional state of patients with CRC or NSCLC.^282,294^ In a variety of cancers, a high level of TSC22D3 is considered an indicator of poor prognosis.^282^ Indeed, NSCLC patients treated with corticosteroids show a poorer response to PD-1/PD-L1 blockade.^295^ It is currently unclear whether this is due to the immunosuppressive effects mediated by corticosteroids or simply because advanced cancer patients are more susceptible to get a large amount of corticosteroids for palliative therapy. Though GCs can impair immune regulation of tumors, they are not likely to impact the potency of PD-1 treatment of mouse models with intracranial glioma, possibly due to the unique immunoprivileged TME.^296^ In a mouse model of mesothelioma, dexamethasone was discovered to facilitate lymphocyte depletion within the bloodstream, except for TIME that could elucidate its substantial adverse effect on the advantages of weak chemotherapy combined with immunotherapy.^297^ However, GC treatment did not significantly inhibit the therapeutic effects in mice with mesothelioma undergoing effective cancer treatment.^297^ Thus, the impact of GCs on tumor immunosurveillance and therapeutic efficacy depends on their accessibility within TME, the anatomical location of the tumor, and the immunogenicity of the cancer therapy.

### CAs

CAs, such as DA, have a complex role in the immune system and are generally considered immunosuppressive factors.^272,298^ However, they can also stimulate the immune system under certain conditions.^299,300^ Aversive stimuli can enhance the release of DA within the mesolimbic system of mice and humans,^268,301^ while chronic psychosocial stress can suppress dopaminergic activity.^302^ Notably raised DA concentrations of blood reflect the stress responses associated with malignancy progression. For lung cancer, the increase of circulating DA effectively prevents T cell expansion and toxicity through DRD1.^303^ In mouse models with melanoma, DA has been demonstrated to be immune-suppressing as well. Cross-presentation of tumor antigens by DCs and effector T cell responses can be enhanced by inhibition of DRD3.^304^ However, DA signaling is beneficial for antitumor immunity in some cases. Such as, in mouse models of lung cancer, the activation of DRD2 on MDSCs reduces their tumor infiltration.^305^ Moreover, DA targeting DRD1 reduced nitric oxide production of MDSCs, subsequently reversed local immune suppression of mice with NSCLC and melanoma.^306^ These findings highlight the dualistic nature of CAs in modulating immune responses and suggest that their effects may depend on the context, the specific receptors involved, and the type of immune cells they interact with. Understanding these intricate relationships is essential to creating effective strategies to modulate immune response in cancer treatment.

The immunosuppressive effects of adrenergic signaling in lymphocytes are widely reported. It rapidly and efficiently increased the expression of the orphan nuclear receptor subfamily NR4A, a key mediator of T cell dysfunction.^307,308^ NR4A1 increase inhibited gene expression for some effector proteins, and knockout of *Nr4a1* within mice enhanced the antitumor immunity.^309^ CAs are highly effective in suppressing the initiation of antitumor CD8^+^ T cell responses, which may be attributed to the relatively high expression of β2-ARs in naive T cells.^310^ In a mouse model of B cell lymphoma, long-term use of ISO largely eliminated the efficacy of tumor vaccines targeting NKT cells and immunotherapies targeting co-stimulatory or coinhibitory molecules. This is primarily as a result of the decline in cytotoxic T cell activities.^142^ Advanced study indicates that prolonged adrenal signaling activation of CD8^+^ T cells inhibited metabolic adaptation, resulting in damaged mitochondrion and affecting glucose metabolism.^311^ In addition, a significant increase in CAs caused by stress favored myeloid cell-driven immunosuppression. β2-AR signaling has been shown to enhance the M2-type macrophage activation in mammary cancer, which subsequently accelerates their infiltration in the tumor and promotes metastasis.^140,312^ This signaling additionally contributed to the accumulation of MDSCs in TIME and increased Arg1 and PD-L1 levels.^138^ In individuals with prostate tumors, particularly those with depression manifestations, NE-induced neuropeptide Y has been shown to be related to a higher intensity of CD68^+^ TAMs, which inhibited antitumor immunity locally.^313^ These findings underscore the complex interplay between the nervous system, the immune system, and cancer progression, highlighting potential targets for intervention to enhance the efficacy of cancer immunotherapies.

The genetic deletion or pharmacological inhibition of β2-ARs, using agents such as ICI118.551 or propranolol, rather than blocking β1-ARs with metoprolol,^143^ has been shown to enhance the efficacy of targeted-PD-1 treatment of mice with melanoma and mammary cancer.^314,315^ Similarly, the blockade of stress-induced β2-ARs significantly improved the abscopal effect of ionizing radiation therapy in mice with melanoma, breast cancer, and CRC, by boosting antitumor immunity.^316^ The combination of β2-AR inhibition and local injection of TLR2 agonists has been shown to amplify the efficiency of tumor antigen-loaded DC vaccines in mice with thymoma.^317^ It has been supported that pharmacologic inhibition of β-AR improved antitumor immunity of cancer patients in clinical trials recently. In a study involving participants with breast cancer, preoperative application of nonselective β-AR antagonist propranolol decreased intratumoral mesenchymal polarization, promoted M1-type macrophages along with conventional type DCs infiltration in TME, as well as enhanced the aggregation of CTLs within the tumor^318^ (Fig. 6). In another randomized, placebo-controlled phase II clinical trial, the perioperative inhibition of β-ARs and cyclooxygenase-2 (COX-2) in breast cancer patients significantly suppressed the expression of genes related to epithelial-mesenchymal transition. It also decreased the activation of transcriptional regulators that promote metastasis or inflammation.^319^ This combined therapy also enhanced the production of IL-12, IFN-γ and CD11a, inhibited the secretion of IL-6 and C-reactive protein, and reduced the mobilization of CD16^−^ monocytes.^319^ These findings suggest that targeting the adrenergic signaling pathway may be a promising strategy to improve the effectiveness of cancer immunotherapies and that the timing and combination of such interventions could be critical in optimizing treatment outcomes.

In a non-randomized, non-blinded clinical trial, non-indicated administration of propranolol upon cancer diagnosis was found to reduce the chance of relapse for melanoma.^320^ In another study, metastatic melanoma participants using nonselective β-AR inhibitors, instead of selective β1-AR antagonists, had an extended overall survival period after receiving therapy targeted with IL-2 plus CTLA-4 and/or PD-1 checkpoint inhibitors.^321^ The β-AR antagonist deserves to be investigated further, enabling it to be used more precisely in specific TME. Interestingly, the activation of dopaminergic input from the ventral tegmental area to the medial prefrontal cortex using optogenetic methods was found to alleviate anxiety symptoms induced by chronic stress, reverse the increase in cytokines associated with tumor growth and progression (such as VEGF, bFGF, and IL-6), and significantly slow the advancement of breast cancer.^260,322^ These findings suggest that not only pharmacological interventions but also novel approaches like optogenetics might play a role in modulating the stress response and potentially impacting cancer progression. The integration of such strategies could open new avenues for cancer treatment and further underscores the complex interplay between the nervous system, the immune system, and cancer biology.

### Other stress-associated immunomodulatory molecules

The impact of stress on tumor immunity is an area of growing investigation. For example, acute stress has been linked to immune evasion in tumors through increased kisspeptin and its receptor GPR54 levels on immune cells, which affects T cell function via the NR4A1-ERK5 pathway.^323^ Additionally, endocannabinoids like anandamide are involved in stress response and have been found to negatively affect CTLs via the cannabinoid receptor 2, related to poorer survival in cancer patients.^324,325^ These insights reveal the complex interplay between stress responses and the immune system’s capacity to detect and fight cancer, potentially leading to new therapeutic approaches targeting stress pathways to boost cancer immunotherapy effectiveness. Recognizing the influence of these molecules on TME and immune function may offer fresh perspectives on the links among stress, immunity, and cancer development.

During sustained stress, the parasympathetic nervous system’s stress-reducing functions, including vagal nerve activity as well as synthesis of Ach, are frequently inhibited.^326^ However, these parasympathetic reactions enhance the antitumor immune response, suggesting new therapeutic opportunities. Agonists of α7-AChR have shown the effects of anti-inflammation, antiproliferative, and tumor-suppression in both mice and humans.^295^ Nicotine, a nicotinic ACh receptor agonist, can boost DC activation and CTL responses, improving the efficacy of DC-based antitumor vaccines.^327^ Additionally, diaphragmatic electrical stimulation in mice increased TFF2 secretion by Tms, which can inhibit MDSC expansion and reduce the carcinogenic potential in mice with inflammatory colon cancer.^150^ This suggests that cholinergic/parasympathetic stimulation could complement adrenergic/sympathetic inhibition in cancer treatment strategies. The possibility of combining cholinergic activation with adrenergic inhibition for enhanced tumor control through localized immune responses warrants further investigation.

### Stress-induced metabolic reprogramming of immune cells

Stress is able to modify metabolism processes within immune cells that circulate in the blood or reside in tissues.^328^ There is rising proofs that altered metabolism and metabolites will influence the survival and function of leukocytes,^329^ through disturbing their intracellular energy provision, molecule synthesis, signaling pathways, as well as PTMs.^330^ GCs or CAs can robustly transfer glucose and lipids into blood, supporting the body’s increased demand for energy and biomolecules.^331^ They are also related to metabolism changes within immune cells, that in turn affect the responses to tumors.^332–336^ In mice with mammary cancer, β2-adrenergic signaling significantly promoted immunosuppression of MDSCs and TAMs through metabolism alterations,^139,337^ that enhanced oxidative phosphorylation, lipids oxidation, and production of prostaglandin E2 (PGE2), however, decreased glycolysis in tumor-infiltrating MDSCs.^139^ The immunosuppressive response of β2-AR signaling in TAMs can be intensified by upregulation of the COX-2/PGE2 axis.^337^ T cell antitumor immune function can be impaired by metabolic disturbances, for instance mitochondria damage and low energy availability.^338,339^ In mice with melanoma and CRC, persistent stress diminished glycolytic pathway as well as oxidative phosphorylation in T cells through β2-AR signaling, leading to T cells exhausted in the TIME.^340^ In mice with sarcoma, GCs reduced low-affinity Tm through inhibiting lipid metabolic activities, resulting in impaired efficacy of immune checkpoint blockade.^341^ It has been reported that chronic stress-induced adrenaline in mice and human breast tumors upregulates LDHA and enhances glycolysis.^342^ LDHA increase as well as excessive lactate within cancer favor immune escape led to poor prognosis of melanoma,^343^ that associates with decreased levels of the activated T cell nuclear factor (NFAT) and IFN-γ within CTLs.^343^ Upon elevation of GCs, CAs, as well as 5-HT, neutrophils can speedily secrete several inflammation mediators such as S100A8 and S100A9, thereby facilitating tumor immune evasion,^295,344^ Additionally, under the state of stress, neutrophils undergo fatty acid oxidation, which promotes the relapse of dormant pulmonary and ovarian malignancies.^344^ Understanding these complex interactions between stress, immune cell metabolism, and cancer progression is crucial for developing strategies that may enhance the effectiveness of immunotherapies and improve cancer treatment outcomes.

### Stress-mediated protein modification

PTMs are able to modulate the immune reactions, which are usually mediated by biologically active metabolites of metabolic reprogramming in immune cells.^345^ It has been indicated that CAs promote lactate production via anaerobic glycolysis.^342,346^ Within TME, elevated lactate derived from external and internal origins in TAMs has been verified recently,^347^ which in accordance with increasing lysine lactylation on histones and transition to an M2-polarized state,^347^ which can contribute to a tumor-promoting microenvironment.

The regulatory impact of PTMs on immune responses has been well-documented, yet the connections to stress and stress-induced inflammation mediators are just starting to be uncovered.^345^ O-β-N-acetylglucosamine (O-GlcNAc) transferase catalyzes the addition of O-GlcNAc to the serine or threonine residues of target proteins.^348^ O-GlcNAcylation is known to enhance the function of lymphocytes, promote neutrophil stimulation, modulate inflammation responses of macrophages, as well as hinder the cytotoxicity of NK cells.^348^ Notably, the release of GAs or CAs induced by stress usually leads to hyperglycemia and immune dysregulation.^349,350^ Hyperglycemia has been reported to enhance O-GlcNAcylation of macrophages, which favors M2-type polarization, and afterward aids immune escape within the CRC.^351^ Palmitoylation, the covalent attachment of palmitic acid to the cysteine residues of target proteins, is primarily catalyzed by zinc finger DHHC domain-containing palmitoyl acyltransferases (ZDHHC),^308,351^ which exhibit the regional and cell-specific patterns of expression within murine cerebral structure.^352^ The palmitoyl proteome of neural synapses is susceptible to the effects of chronic stress.^353^ Degradation of PD-L1 is inhibited by ZDHHC3-induced palmitoylation.^354^ ZDHHC3 blocking can reduce the PD-L1 expression of tumor cells, thereby enhancing antitumor immunity.^354^ Thus, the fluctuations in stress-induced metabolites and PTMs could facilitate the precise modulation of cancer immunity. Understanding these complex interactions can provide insights into the development of targeted therapies that modulate the TIME and improve the efficacy of cancer treatments.

### Stress management affects cancer immune surveillance

Alleviation of pressure through physical activity and mindfulness- or cognitive-behavior-based methods, for instance, yoga, tai chi, or meditation, has been associated with immunological changes, including reduced inflammation, increased responses of Th1 cells, and enhanced activity of NK cells.^355^ Psychological interventions have been found to significantly notably minimize the chance of death or relapse within non-metastatic breast cancer patients when they were supplied before surgery and adjuvant therapy,^356^ as well as enhance life quality and personal happiness for individuals with cancer.^357^ It is noteworthy that CAs induced by running (not “psychological stress”) can activate YAP/TAZ phosphorylation within mammary carcinoma, subsequently hindering tumor growth of immunodeficient mice,^358^ which suggests that the transient peak of CAs, intrinsic opioids, or myokines after physical activities could inhibit cancer growth.^359–361^ A study on liver cancer demonstrated that eustress, or positive stress, can enhance the sensitivity of mice to liver cancer immunotherapy. Eustress activates the peripheral sympathetic nervous system in mice, leading to increased levels of NE and epinephrine (EPI) in serum and tumor tissue. The increase in NE and EPI activates β-ARs signaling in tumor cells and tumor-infiltrating myeloid cells. Activation of β-ARs leads to a direct reduction in CCL2 expression in tumor cells and immune cells, reducing the infiltration of TAMs and MDSCs in the TME and enhancing the infiltration and function of CD8^+^ T cells. This increases sensitivity to ICIs, such as anti-PD-L1 therapy.^362^ Specifically, some psychotropic medications may exhibit immunostimulatory properties (e.g., affecting the intensity of lymphocytes in the tumor). Psychotropic inhibitors and stimulants used in patients with triple-negative breast cancer are associated with a higher rate of pathological complete response.^363^ This study underscores the multifaceted impact of stress management on immune function and its potential to influence the effectiveness of cancer therapies. Future studies need to figure out the cell subpopulations and receptors in the TME that interact with them and whether they are specific to a particular tumor type. The exploration of these pathways could lead to novel strategies for enhancing the body’s natural defenses against cancer and improving treatment outcomes.

## The brain-gut axis and tumor immunity

**Fig. 7:**
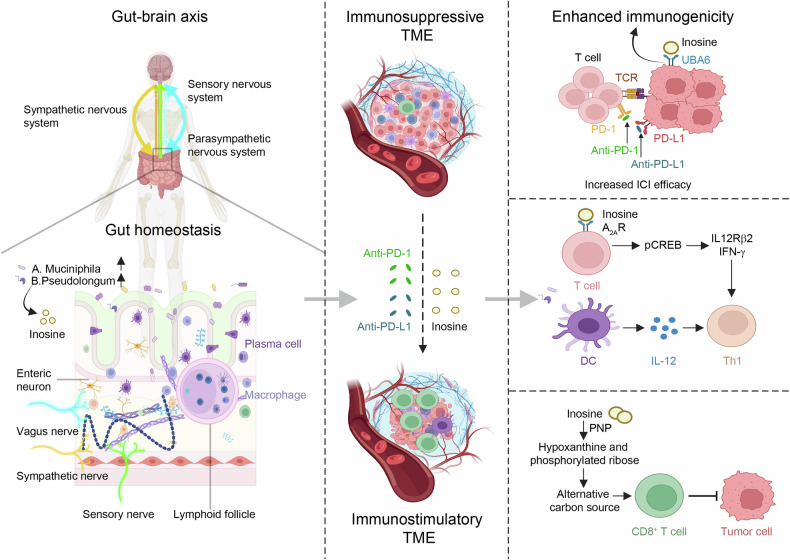
Neuro-immune regulation maintains gut homeostasis and enhances the efficacy of cancer immunotherapy. The gut and brain communicate through the gut-brain axis (GBA). Brain regulates the function of the gut through peripheral sympathetic, parasympathetic, and sensory nerves, as well as the extensively distributed enteric nervous system (ENS). These regulatory functions include gastrointestinal motility, digestive secretion, immune function, blood flow, and nociception. The nervous and immune systems collaborate to maintain intestinal homeostasis, which is conducive to the growth of probiotics. A. muciniphila and B. pseudolongum are canonical probiotics known for their capacity to produce inosine, which can facilitate the efficacy of ICIs. Inosine enhances the capacity of tumor cells to present tumor-associated antigens, thereby facilitating the recognition and subsequent elimination of these cells. Inosine also plays a role in promoting the activation of immune cells. Specifically, engaging the A_2A_ receptor (A_2A_R) on T lymphocytes, through the inosine-A_2A_R-cAMP-PKA signaling cascade, inosine triggers the phosphorylation of cAMP response element-binding protein (pCREB), which in turn upregulates the transcription of IL12Rβ2 and IFN-γ, driving the differentiation and accumulation of Th1 cells within TME. Additionally, inosine serves as an alternative carbon source for CD8^+^ T cells, particularly under conditions where glucose availability is scarce. This role of inosine helps to alleviate energetic constraints on CD8^+^ T cells within TME, potentially enhancing their antitumor activities. This figure was created with BioRender (https://biorender.com/)

The gut and the CNS communicate via the gut-brain axis, which is a bidirectional network between the brain and the gut microbiota.^364^ It is well-established that the brain regulates the function of the gut through peripheral sympathetic, parasympathetic, and sensory afferent and efferent neurons, as well as the extensively distributed enteric nervous system.^365^ These regulatory functions include gastrointestinal motility, digestive secretion, immune function, blood flow, and nociception.^365–368^ Dysfunctions of the autonomic nervous system (ANS) lead to an imbalance in the regulation of the gut-brain axis, thereby causing dysbiosis.^369^ Interestingly, there is evidence suggesting that the gut can influence tumor immunity through the synthesis and metabolism of microbial-derived products. Short-chain fatty acids (SCFAs) and amino acid metabolites, such as glutamate, glutamine, and tryptophan. These microbial metabolites can act as signaling molecules, influencing immune responses and potentially contributing to the modulation of antitumor immunity (Fig. 7).

### Dysbiosis and TIME

Dysbiosis, the dysfunction of the gut microbiota due to an imbalance in the normal gut flora, is recognized as a risk factor for a variety of diseases. Metagenomic sequencing analysis has revealed that an imbalanced gut microbiota can contribute to conditions such as irritable bowel syndrome, diabetes, obesity, and cancers like CRC.^370–372^ In fact, dysbiosis has been demonstrated to promote carcinogenesis in CRC.^364,373–376^ Metabolic byproducts resulting from the ecological imbalance of the gut microbiota, such as toxins and cytokines, can lead to inflammation and tumorigenesis, thus contributing to the development of CRC. This suggests that a healthy gut microbiota may play a role in tumor suppression.^377^ Furthermore, recent studies have provided evidence supporting the role of the gut microbiota, in conjunction with the TME, in modulating responses to antitumor therapies.^378–380^

More specifically, regarding brain tumors, the composition and related functions of the gut microbiota appear to be largely controlled by the interaction between the gut and the CNS through the gut-brain axis.^378^ The microbial structure in the context of brain tumors, such as gliomas, pituitary adenomas, and meningiomas, has been studied. It has been found that the microbial composition and structural assembly associated with these tumors are dependent on the type of tumor,^372,381–384^ highlighting the importance of precision medicine. In the brain, dysbiosis and microbiota-associated metabolic products, such as neurotransmitters, SCFAs, and amino acids, can cause changes in the CNS TME, which may be related to tumors.^366,370,374–377^ It was demonstrated that compound K, a metabolite derived from ginsenoside and biotransformed by the gut microbiota, inhibited the migration of glioma cells, which were stimulated by stromal cell-derived factor-1 (SDF-1).^385^ SDF-1 has previously been shown to positively regulate the growth and migration of glioma cells.^386^

SCFAs are involved in maintaining the integrity of the BBB, acquiring the mature phenotype of microglia, and the reactivity to brain injury.^387,388^ In the context of glioma, which is associated with the dysregulation of the gut microbiota, there is an increased permeability of the BBB and immune suppression modulation, including the activity of immune cells such as microglia.^387,388^ In glioma-bearing mice treated with antibiotics, subsequent dysbiosis of the gut leads to a reduction in cytotoxic NKT cells and alterations in the expression of inflammatory proteins in microglia associated with increased tumor growth, indicating a change in the antitumor activity of immune cells.^382^ Furthermore, microglia have been shown to promote the progression and angiogenesis of glioma through TREM2 activation,^389^ a pathway known to be upregulated by SCFAs.^390^

In metastatic cancer, the activation of receptors for SCFAs (such as propionate and butyrate), specifically free fatty acid receptors (FFAR2 and FFAR3), inhibits the invasive phenotype of breast cancer cells, thereby suppressing metastasis.^373,391^ These receptors play a crucial role in modulating inflammation and the production of leptin,^374^ stimulating insulin secretion in response to glucose,^375^ the secretion of glucagon-like peptide-1,^376^ and the production of peptide YY.^378^ Multiple studies have indicated that FFAR2 and FFAR3 are involved in tumor suppression. In CRC, the expression of FFAR2 is significantly reduced, and propionate, a short-chain fatty acid, has been shown to play a significant role in reducing cell proliferation and inducing apoptosis.^374,380,381,392,393^ These findings suggest that SCFAs and their activation of FFAR2 and FFAR3 receptors could support a model that may aid in the study of the progression of certain cancers.

### The gut microbiota influences the efficacy of cancer immunotherapy

The gut microbiota can influence the efficacy of cancer immunotherapy.^394–403^ The manipulation of the gut microbiota through methods like fecal microbiota transplantation (FMT),^397,404–408^ probiotics,^409^ engineered microbiota, and specific microbial metabolites, such as inosine, can enhance the effectiveness of immunotherapy (Fig. 7). These findings offer new insights into the development of novel cancer treatment strategies based on the microbiome and emphasize the importance of precision medicine.^397,410–412^ Conversely, the disruption of gut microbiota balance through the use of antibiotics may affect the efficacy of ICIs.^408,409,413–417^ This highlights the delicate relationship between the gut microbiota and the immune system and suggests that maintaining a balanced microbiota is crucial for the success of immunotherapy in cancer treatment. Understanding the complex interactions between the gut microbiota and the immune system is essential for developing strategies that can optimize the response to immunotherapy. This may involve the identification of key microbial species or metabolites that promote a favorable microenvironment for immune cell function and the development of interventions that can modulate the gut microbiota to enhance the therapeutic outcomes for cancer patients.

## The impact of tumor immunity on the nervous system

### Perineural invasion (PNI), nerve injury and neuritis, cancer, and therapy induce pain

PNI is characterized by the infiltration of tumor cells into adjacent nerves, which can lead to metastatic spread, nerve compression, and pain generation. This phenomenon is associated with a poor prognosis in various cancers, including pancreatic, oral, colorectal, adenoid cystic carcinoma, HNSCC, and prostate cancer.^418^ TAMs contribute to PNI by releasing cytokines such as IL-6, TNF, CCL5, and CCL18 in response to hypoxic or high-lactate environments, leading to peripheral inflammation and the release of neurotrophic factors, growth factors, and MMPs, which facilitate tumor growth and invasion.^419^ When nerves are initially damaged by cancer cells, neurons and Schwann cells produce artemin/GFRα3 to repair the nerves.^420^ However, the abundance of artemin can attract more cancer cells to the site of injury. Infiltrating tumor cells can cause nerve damage and release CCL5, inducing an inflammatory response that promotes the migration of cancer cells expressing CCR5 to the injured nerve, thus enhancing PNI.^421^ Tumor-derived factors and inflammatory mediators can also activate peripheral sensory fibers, leading to the release of SP that promotes tumor growth. SP enters the tumor, activates NK-1R in cancer cells, and through Src (EGFR, HER2), activates growth factor receptors and the MAPK pathway, including ERK1/2. This leads to increased mRNA expression of MMP2, MMP9, VEGF, and VEGFR, stimulating mitosis, cell proliferation, and preventing apoptosis.^422^ PNI established connections with distinct brain regions in head and neck cancer. Activation of these neural circuits altered mouse behavior, including reduced nest-building, increased latency to eat a cookie, and decreased wheel running. Disrupting the connecting nerves through genetic or pharmacological means improved all behavioral outcomes in tumor-bearing mice.^423^ The study suggests that blocking neural connections between tumors and the brain may enhance mental health in cancer patients, although further investigations are needed to establish this link.

In addition to tumor cell invasion, immune cells' infiltration can lead to neuritis. For example, in PDAC, pancreatic nerves are infiltrated by various immune cells, including mast cells, which are associated with the intense abdominal pain experienced in PDAC. TME, characterized by hypoxia and acidity, induces apoptosis in the tumor and peripheral tissues, releasing cellular debris and chemotactic factors that attract inflammatory cells and promote inflammation. The tumor itself triggers immune responses, leading to the aggregation of inflammatory cells and severe inflammation, which promotes tumor growth and infiltration into neural tissues.^424^ Mitochondrial dysfunction and altered glucose metabolism are typical changes in TME and may be related to the etiology of neurodegenerative diseases and neural injury.^425^ Oxidative stress can induce chronic neuroinflammation, leading to a functional shift in astrocytes from neurotrophic to neurotoxic, releasing more lactate to enhance their energy support for neurons.^426^ Following partial peripheral nerve injury, sustained demyelination can promote neural sprouting.^427^ In cancers such as PDAC, there is an increase in nerve density and size, altering the distribution of nerves within tumors to facilitate signal exchange between cancer cells and nerves. This can be interpreted as pathological neural plasticity induced by tumor-derived factors.^428^

Cancer-related pain often results from the invasion of sensory neurons by cancer cells, which can directly elicit pain sensations.^429^ The subsequent neuroinflammatory response can exacerbate pain, and there is a potential link to side effects from therapeutic interventions.^430,431^ PDAC, known for its intense pain, frequently involves PNI, which is associated with poorer survival outcomes and a diminished quality of life. PNI contributes significantly to cancer pain because of the similarity among signaling molecules implicated in PNI and those associated with pain signaling pathways.^429^ Cancer pain can present in various forms, including persistent, intermittent, aching, sharp, or neuropathic.^432^ Within TME, a spectrum of inflammatory mediators can bind to specific receptors on peripheral sensory neurons, converting into pain signals that are conveyed to higher neural centers and perceived as pain.^433^ Nociceptors, including C-low threshold mechanoreceptors expressing Piezo2 receptor, can be activated through mechanical stimuli, leading to heightened sensitivity to external stimuli and amplifying the perception and response to pain.^434^

The molecular and cellular basis of cancer pain is complex, impacting various levels of the pain neurocircuitry. Algesic mediators within TME sensitize peripheral sensory neurons, contributing to hyperalgesia and allodynia. These mediators may originate from the tumor and immune cells and include inflammatory factors, cytokines, chemokines, colony-stimulating factors, and neurotrophic factors.^112^ Changes in TME, such as elevated levels of extracellular ATP and protons, can prompt the production of these mediators. These mediators exert their effects through multiple mechanisms, including direct interaction with receptors or ion channels involved in detecting and signaling noxious stimuli, such as protons through TRPV1 and ASIC receptors.^435^ They can also induce the sensitization of these receptors/channels by activating kinases, which phosphorylate TRPV1, leading to its sensitization, or by upregulating the expression of these receptors/channels. Nerve growth factor (NGF), by binding to its TrkA receptor, can induce high expression of TRPV1 and ASIC3, along with neurotransmitters that regulate nociceptor excitability, such as SP, CGRP, BDNF, and various sodium and calcium channels.^435^ The sensitization of TRPV1 can also occur through interplay with other receptors, including P2X3 and NMDA.^436,437^ Inflammatory mediators produced by immune cells contribute to the exacerbation and maintenance of pain. Pro-inflammatory cytokines, including TNF-α, IL-1β, and IL-6, as well as other inflammatory mediators like PGE2, play a role in cancer pain across various cancer types.^438,439^ Therapies targeting pro-inflammatory molecules are becoming potential solutions for managing pain. Nonsteroidal anti-inflammatory drugs (NSAIDs) are frequently employed in clinical practice to provide additional pain relief in conjunction with more potent analgesics.^440^

Cancer-related pain may arise from increased tissue innervation due to ectopic neural sprouting as well. In TME, neurotrophic factors such as NGF, BDNF, GDNF, and VEGF are released, leading to neural sprouting.^441^ Molecules engaged in axon guidance, including those from the ephrin and netrin families, contribute to neural sprouting within tumors. This disordered sprouting leads to an overall elevation in nerve fiber intensity and the formation of neuroma-like structures.^435^ Neural sprouting can disrupt the physiological separation of sensory and sympathetic nerves, with the activation of sensory neurons by sympathetic nerves potentially leading to movement-induced pain.^442,443^

The aim of cancer pain management is to reduce suffering, enhance quality of life, and minimize side effects. Treatment strategies include pharmacological therapies (opioids, NSAIDs, antidepressants, anticonvulsants), non-pharmacological interventions (physical, psychological therapies, neurostimulation), and tumor-directed treatments (surgery, chemotherapy, radiotherapy, immunotherapy). Personalized treatment plans should be adjusted based on disease progression and treatment responses.^444^

### Paraneoplastic neurological syndromes (PNS)

PNS refers to a group of neurological symptoms that occur in cancer patients. These symptoms are not caused by direct tumor invasion, distant metastasis, infection, metabolic abnormalities, or side effects of treatment, but are mediated by the body’s immune response to antigens expressed on tumor cells, which are also expressed in the host’s nervous system.^445^ Antigens released after tumor cell apoptosis are presented to Th cells in peripheral lymph nodes by antigen-presenting cells. Subsequently, CD4^+^ helper T cells activate antigen-specific B cells to become antibody-producing plasma cells. These antigens are divided into cell surface antigens and intracellular antigens.^446–448^ The mechanisms of disease targeting these two types of antigens differ. Antibodies against intracellular antigens are not directly pathogenic but serve as biomarkers for cytotoxic T cell-mediated tissue damage.^449^ CD8^+^ T cell infiltration has been found in the neural tissues of patients with antibodies against intracellular antigens.^450–452^ In contrast, antibodies against cell surface antigens bind in vivo, and numerous studies have elucidated the direct pathogenic mechanisms.^453,454^ For example, antibodies against AMPA-R, NMDA-R, and GABA-Rs cause neuronal dysfunction by receptor cross-linking and internalization, leading to a reduction in cell surface receptor density.^450,455–458^ On the other hand, GABAR antibodies directly impair receptor function without causing internalization.^459^ Aquaporin-4 antibodies mediate antigen internalization and complement-induced cytotoxicity, while LGI1 antibodies affect protein-protein interactions with their receptor ADAM22.^460,461^

The clinical presentation of neurological disorders can vary depending on the specific antibodies induced by the particular tumor.^462,463^ These disorders often manifest as a diverse range of neurological symptoms, such as paraneoplastic encephalitis,^464^ paraneoplastic cerebellar degeneration,^465^ neuritis,^466–470^ and myasthenia gravis syndrome^462^ (Table 1).

**Table 1:** Cancer types associated with paraneoplastic neurologic disorders

Cancer	Antibody	Antigen	Antigen location	Neurologic syndromes
SCLC, thymoma, NEC, neuroblastoma^462^	Anti-Hu (ANNA-1)	HuD and related nuclear proteins	Intracellular	Encephalitis, myelitis, encephalomyelitis, peripheral neuropathy
Ovarian, breast cancer^465^	Anti-Yo (PCA-1)	CDR2	Intracellular	Cerebellar degeneration
Breast, ovarian cancer, SCLC^446,447^	Anti-Ri (ANNA-2)	NOVA	Intracellular	Cerebellar ataxia, opsoclonus, brainstem encephalitis, Parkinsonism
Hodgkin lymphoma^453^	Anti-Tr (DNER)	DNER	Intracellular	Cerebellar degeneration
SCLC, thymoma^454^	Anti-CV2/CRMP5	CRMP5	Intracellular	Encephalitis, myelitis, encephalomyelitis, cerebellar degeneration, optic and peripheral neuropathy
Testicular germ cell tumors, lung, breast cancers^452^	Anti-Ma1, Anti-Ma2	PNMA1, PNMA2	Intracellular	Limbic encephalitis, brainstem encephalitis, cerebellar degeneration
SCLC, gynecologic Cancer^445^	Anti-Recoverin	Recoverin	Intracellular	Retinopathy
Testicular (seminoma), teratoma^456,464,472^	Anti-Kelch-like Protein	Kelch-like Protein	Intracellular	Ataxia, brainstem encephalitis, diplopia, hearing loss, and vertigo
Breast cancer, SCLC^448,449^	Anti-Amphiphysin	Amphiphysin	Intracellular	Stiff person syndrome, Encephalomyelitis
Ovarian teratoma, Testicular germ cell tumors^455,489^	Anti-NMDA-R	NMDA-R	Extracellular	Limbic encephalitis
Breast cancer, thymoma, SCLC, Ovarian cancer^457^	Anti-AMPA	AMPA	Extracellular	Limbic encephalitis
Hodgkin lymphoma, Thymoma^458^	Anti-GABAA	GABAA	Extracellular	Refractory status epilepticus
SCLC^459^	Anti-GABA-B	GABAB	Extracellular	Limbic encephalitis with seizures, opsoclonus, ataxia
Hodgkin lymphoma, Prostate, Cutaneous T Cell lymphoma^463^	Anti-mGluR1	mGluR1	Extracellular	Cerebellar degeneration
SCLC^476^	Anti-P/Q VGCC	P/Q VGCC	Extracellular	LEMS, cerebellar Degeneration
Thymoma^492^	Anti-AChR	AChR	Extracellular	Myasthenia gravis, autonomic neuropathy

*CDR2* cerebellar degeneration related protein 2, *NOVA* neuro-oncological ventral antigen, *DNER* Delta/Notch-like epidermal growth factor-related receptor, *CRMP5* collapsin response mediator protein 5, *PNMA* paraneoplastic ma antigen, *NMDA-R* N-methyl-D-aspartic acid receptor, *AMPA* α-amino-3-hydroxy-5-methyl-4-isoxazolepropionic acid, *mGluR1* metabotropic glutamate receptor 1, *P/Q VGCC* P/Q-type voltage-gated calcium channel

In a study on 26 ovarian cancer tumors associated with Yo-paraneoplastic cerebellar degeneration (Yo-PCD), the immune contexture of the tumors and the genetic status of two tumor-associated antigens (Yo antigens), CDR2 and CDR2L, were analyzed. The study found that Yo-PCD tumors had a richer infiltration of T and B cells compared to control tumors, occasionally forming tertiary lymphoid structures with CDR2L protein deposits. Immune cells were primarily located near apoptotic tumor cells, indicating an immune attack on the tumor. Moreover, 65% of Yo-PCD tumors had at least one somatic mutation in the Yo-antigen genes, mainly missense mutations, compared to the control group.^462^ In patients with breast cancer combined with Yo-PCD, these breast cancers were predominantly aggressive, HER2-positive, and hormone receptor-negative, with early lymph node metastasis. All Yo-PCD breast cancers carried at least one genetic variation leading to the overexpression of Yo antigens.^471^ Anti-Kelch-like protein 11 (KLHL11) paraneoplastic encephalitis is commonly seen in testicular germ cell tumors.^464,472^ A retrospective identification of 31 KLHL11 IgG-positive cases was conducted, and human leukocyte antigen typing and assessment of KLHL11-specific T cell responses were performed. The study found no increased activation of CD4^+^ and CD8^+^ T cells in response to KLHL11 antigen stimulation in healthy controls compared to KLHL11 IgG-positive patients.^472^ In SCLC, paraneoplastic encephalitis associated with anti-Hu, GABABR, and anti-voltage-gated calcium channels has been observed.^464,473–476^ In patients with anti-GABABR PNS, there is a common gain of chromosome 5q, containing KCTD16, while losses are often seen in anti-Hu and control patients.^25^ Transcriptome analysis revealed the specific overexpression of KCTD16 in anti-GABABR PNS.^25^ In patients with late-onset paraneoplastic encephalitis associated with prostate adenocarcinoma, these patients exhibited psychiatric symptoms related to autoimmunity against the DRD2, indicating that in some cases, tumors may trigger an autoimmune response against dopamine receptors, leading to neurological symptoms.^477^ These studies show that tumor-mediated immune responses can damage the nervous system, with diverse manifestations that may precede the diagnosis of the tumor.

In addition to immune responses mediated by tumor-associated antibodies and antigens that can lead to PNS, the binding of cytokines to receptors can also cause neurologic dysfunction. For instance, the binding of GDF15 to GFRAL receptors in the brainstem can induce anorexia, leading to reduced food intake and subsequent cachexia in cancer patients.^478–481^ GDF15 has been found to be expressed in tumor cells, TAMs, and fibroblasts,^482,483^ and its levels are elevated in the serum of cancer patients or mice.^481,484–486^ Neutralizing GDF15 or pharmacologically inhibiting it can reverse anorexia and weight loss in mice.^481^ However, it has been found that neutralizing GDF15 with a potent monoclonal antibody (mAB2) did not improve anorexia and weight loss caused by low-dose lipopolysaccharide-induced inflammation.^487^ Therefore, further studies are needed to assess the efficacy of targeting GDF15 therapies.

The treatment of paraneoplastic syndromes encompasses neuroimmunological therapies, cancer-specific treatments, and symptomatic therapies. Diseases associated with cell surface antibodies respond better to immunotherapy than other paraneoplastic diseases, while those associated with intracellular antineuronal antibodies respond poorly to immunotherapy.^488^ Commonly used medications in immunotherapy include corticosteroids, intravenous immunoglobulins, immunosuppressive agents (such as cyclophosphamide), and plasmapheresis.^489^ In patients with anti-NMDA-R encephalitis, rituximab is often used early in the course of severe neurological disease.^489^ Under the guidance of oncology and surgery, cancer treatment may coincide with the stabilization or improvement of neurological symptoms. Some patients with anti-NMDA-R encephalitis have significant improvement in neurological symptoms after the removal of ovarian teratomas. To improve neurological symptoms, various treatments are employed for conditions such as myasthenia gravis (pyridostigmine),^490^ Parkinson’s syndrome (levodopa), dystonia (benztropine or botulinum toxin), epilepsy (Sodium Valproate),^491^ myoclonus (benzodiazepines).^490,492^

### ICIs induced neurological disorders

ICIs have revolutionized cancer therapy, but their systemic activity can lead to a broad range of immune-related adverse effects on the nervous system (nirAEs),^493,494^ which include peripheral neuropathy,^495,496^ myositis,^496–498^ myasthenia gravis,^496,499–502^ Guillain-Barré syndrome,^503–505^ encephalitis, and other CNS disorders.^506^ Although the incidence is relatively low (about 1%–6%), the potential severity and the possibility of needing to interrupt cancer treatment make these side effects particularly significant. Despite the exact mechanisms of neurotoxicity induced by ICIs not being fully understood, the enhancement of immune responses mediated by CD8^+^ T cells through the blockade of tumor suppressive signals appears to play a decisive role.^507–509^ Concurrently, CD4^+^ T cells are also of significant importance. In a patient with metastatic melanoma who developed fatal encephalitis following anti-PD-1 therapy, spatial and multi-omic analyses revealed a significant enrichment of activated memory CD4^+^ T cells in the inflamed brain region. A highly oligoclonal T cell receptor (TCR) repertoire was identified and localized to activated memory cytotoxic (CD45RO^+^GZMB^+^Ki67^+^) CD4^+^ T cells.^369^ In addition, mechanisms such as the downregulation of Tregs, the cross-presentation of neoantigens, epitope spreading, genetic predisposition, and alterations in the microbiome are involved in this process.^510,511^

Current treatment recommendations include discontinuation or suspension of immunotherapy and the use of high-dose corticosteroids, intravenous immunoglobulins, plasmapheresis, or other immunosuppressive drugs in refractory cases.^495,508,512^ However, further structured study is needed to better understand and optimize the clinical management of neuro-immune-related adverse events.^507^ The recognition of these neurological side effects emphasizes the importance of a multidisciplinary approach to cancer care, integrating neurology and oncology to manage the complex interplay between immunotherapy and the nervous system. It also underscores the need for ongoing clinical vigilance and research to improve patient safety and outcomes in the era of immunotherapy.

## The potential of neuroimmunotherapy in cancer treatment

The growing data on neuro-immune bidirectional communication within the TME suggests that targeting the nervous system holds promise as a therapeutic approach. This strategy can strengthen existing treatments as well as enhance the prognosis of patients. In TME, the modulation of adrenergic signaling through physiology, pharmacology, and genetics has shown that reducing adrenergic stress leads to an increase in CD8^+^ T cells infiltration, a lower rate of PD-1 receptor expression,^315,318^ and a better effect of anti-PD-1 treatment.^315^ A preclinical study reported that in mice with fibrosarcoma as well as CRC, the concurrent use of propranolol and anti-CTLA-4 antibody increased T lymphocytes and reduced the infiltration of MDSCs into TME, significantly enhancing the effectiveness of anti-CTLA-4 therapy.^144^

### Clinical trials completed

Clinical trials targeting the neuro-immune axis in cancer were being assessed based on the regulatory effects of drugs on neural signaling (Table 2). These included direct alteration of neural function (e.g., lidocaine) and targeting downstream activities of the nervous system following stimulation and subsequent neurotransmitter release (e.g., propranolol). Phase I clinical trial of combination propranolol and pembrolizumab in locally advanced and metastatic melanoma (NCT03384836) showed no dose-limiting toxicities, and a response rate of 78%. Patients treated had higher levels of IFN-γ, while lower levels of IL-6.^513^ Preoperative β-blockade with propranolol can reduce metastasis biomarkers of breast cancer in a phase II randomized trial (ACTRN12615000889550).^318^ For instance, reduced intratumoral mesenchymal polarization, elevated tumor infiltration of CD68^+^ macrophages, and CD8^+^ T cells were observed in patients treated in early-stage surgically resectable breast cancer. Another clinical trial also showed an increase in tumor-infiltrating CD8^+^ T cells and the expression of GzmB in CRC patients after one week of administration of propranolol (NCT03245554). In addition, the intratumoral level of phosphorylated ERK was down-regulated. However, there was no significant difference in Ki67 or the expression of p-AKT and p-MEK.^514^ This indicates that propranolol suppresses tumor growth by enhancing the infiltration of CD8^+^ T cells into the TME, rather than directly affecting the tumor itself, which is consistent with the aforementioned observation that the application of propranolol in mice with melanoma leads to an increase in tumor-infiltrating CD8^+^ T cells. In the clinical study combined application of propranolol and COX-2 inhibitor in early-stage breast cancer, it was found that various cellular and molecular pathways contributed to metastasis, and the recurrence of disease was suppressed, and tumor-infiltrating monocytes decreased, while tumor-infiltrating B cells increased. Additionally, the expression of CD11a on circulating NK cells was upregulated, whereas the activity of CD16^−^ monocytes was suppressed (NCT00502684).^319^ However, this study involved the combined use of two drugs, and it does not demonstrate that these antitumor immune effects are mediated by propranolol. The concurrent administration of propranolol and COX-2 inhibitor in another clinical trial (NCT00888797) supported the effect of them on the enhancement of antitumor immunity by elevating NK cells in TME.^515^ Although propranolol has shown promising effects in improving the TIME in these clinical studies, the impact on tumor recurrence rates and long-term survival outcomes of patients requires further observation and investigation. Additionally, there is a lack of assessment for β-ARs in cancer specimens. The variation in β-AR levels across different tissues may influence the therapeutic response to β-AR blockers. The patients were not effectively stratified, which may introduce bias in the study outcomes. A potential solution is to assess the expression of β-receptors in cancer specimens pathologically before the commencement of the clinical trial. This approach could help in identifying patients who are more likely to respond to propranolol, thereby enhancing the efficacy of the treatment regimen. Furthermore, the number of patients in these clinical studies is relatively small, necessitating the inclusion of more patients to validate the efficacy of propranolol, and precise stratification of responders and non-responders is essential to maximize the benefit for oncological patients.

**Table 2:** Summary of the completed clinical trials targeting the neuro-immune axis in cancer

Clinical trial	Trial no.	Medication dose, administration route, and schedule	Patient characteristics	Outcomes
Perioperative COX-2 and β-adrenergic blockade improves metastatic biomarkers in breast cancer patients in a phase II randomized trial	NCT00502684	Propranolol was administered orally: (i) 20 mg of immediate release BID during the five days before surgery; (ii) 80 mg of extended release on the morning of surgery and on the evening and morning following surgery; and (iii) 20 mg of immediate release bid thereafter during the five postoperative days.	Women (age 33–70) diagnosed with stage I–III breast cancer were enrolled from three medical centers. 38 participants.	1. Inhibited multiple cellular and molecular pathways related to metastasis and disease recurrence in early-stage breast cancer. 2. Decreased tumor-infiltrating monocytes while increasing tumor-infiltrating B cells. 3. Abrogated presurgical increases in serum IL-6 and C-reactive protein levels, abrogated perioperative declines in stimulated IL-12 and IFN-γ production. 4. Abrogated postoperative mobilization of CD16^−^ monocytes, and enhanced expression of CD11a on circulating NK cells.
Preoperative β-blockade with propranolol reduces biomarkers of Metastasis in breast cancer: a phase II randomized trial	ACTRN12615000889550	40 mg oral propranolol or placebo twice a day starting 7 days prior to the date of surgery. After 3 days of treatment, the dose of study drug was escalated to 80 mg oral propranolol or placebo a day daily until the day of surgery	Age 18–80 years with a diagnosis of surgically resectable primary breast cancer. 60 participants.	1. Reduced intratumoral mesenchymal polarization, 2. Promoted immune cells' infiltration in early-stage surgically resectable breast cancer. 3. Elevated tumor infiltration of CD68^+^ macrophages and CD8^+^ T cells.
Phase I clinical trial of combination propranolol and pembrolizumab in locally advanced and metastatic melanoma: Safety, Tolerability, and Preliminary Evidence of Antitumor Activity	NCT03384836	Pembrolizumab 200 mg every 3 weeks i.v. and progressively increasing propranolol dosing from 10 mg (dose level 1), 20 mg (dose level 2) to 30 mg (dose level 3) twice a day, until 2 years on study or disease progression or dose-limiting toxicities.	Stage III or IV melanoma with good organ function and measurable disease on computed tomography or magnetic resonance imaging scans. 47 participants.	No significant changes in treatment-associated biomarkers, an increase in IFN-γ, and a decrease in IL-6 in responders.
Propranolol suppresses the growth of colorectal cancer through simultaneously activating autologous CD8^+^ T cells and inhibiting tumor AKT/MAPK pathway	NCT03245554	The trial was performed in accordance with clinical practice guidelines. (The specific dosage and administration method of propranolol were not provided). The participants who treated with propranolol and placebo group for 1 week followed by surgical resection.	Patients of 18–70 years old, who have been diagnosed with a stage I–III colorectal cancer requiring surgery, were recruited to receive propranolol or placebo for one week prior to curative resection. 28 participants.	1. Propranolol led to a higher frequency of intratumoral CD8^+^ T cells. 2. Down-regulated the intratumoral level of phosphorylated ERK. 3. No significant differences in Ki67 or the expression of p-AKT and p-MEK were observed.
Perioperative COX-2 and β-adrenergic blockade improves biomarkers of tumor metastasis, immunity, and inflammation in colorectal cancer: A randomized controlled trial	NCT00888797	Drug or placebo for 20 consecutive days, starting 5 days before the tumor resection. Oral etodolac (400 mg twice a day) throughout the treatment period. Propranolol orally infiltration using extended-release formulations at 20 mg twice a day during the 5 days preceding surgery; 80 mg twice on the day of surgery; 40 mg twice a day after the day of surgery for 7 postoperative days; and 20 mg twice a day for the last 7 days.	Patients with a median age of 58 years diagnosed with CRC without known metastatic disease. 34 participants.	1. Reduced tumor infiltrating CD14^+^ monocytes and CD19^+^ B cells. 2. Increased tumor-infiltrating CD56^+^ NK cells.
Neutrophil extracellular trapping and angiogenesis biomarkers after intravenous or inhalation anesthesia with or without intravenous lidocaine for breast cancer surgery: a prospective, randomized trial	NCT02839668	sevoflurane active comparator: sevoflurane + lidocaine; total intravenous anesthesia with propofol using a target-controlled infusion technique (TIVA-TCI); active comparator: TIVA-TCI + lidocaine	Patients with breast cancer. 120 participants.	Decrease in neutrophil extracellular trapping and MMP3 following administration of lidocaine i.v. during surgery. Using lidocaine during cancer surgery of curative intent may decrease recurrence.
Effect of intravenous lidocaine on serum Interleukin-17 after video-assisted thoracic surgery for non-small-cell lung cancer: a randomized, double-blind, placebo-controlled trial	ChiCTR2000030629	Patients in the lidocaine group received a lidocaine bolus of 1.0 mg/kg over 10 min, followed by a continuous infusion at a rate of 1.0 mg/kg/h throughout the surgery. Patients in the control group received the same volume of normal saline instead of lidocaine.	American Society of Anesthesiologists physical status I–III scheduled for elective VATS procedures for early-stage NSCLC 95 participants.	Intravenous lidocaine was associated with reduced serum IL-17 and cortisol following VATS procedures in early-stage NSCLC patients.
Intraoperative lidocaine infusion in patients undergoing pancreatectomy for pancreatic cancer: a mechanistic, multicenter randomized clinical trial	NCT03245346	Lidocaine i.v. (continuous intraoperative infusion of 2 mg kg^−1^ h^−1^, after 1.5 mg kg^−1^ bolus at induction of anesthesia) or saline placebo.	Pancreatic adenocarcinoma amenable to surgical resection with curative intention. 563 participants.	Lidocaine did not alter overall or disease-free survival. Mean intraoperative sufentanil dose was reduced by lidocaine infusion, but postoperative complications and length of hospital stay were similar between groups. Circulating NETs were lower after lidocaine infusion up to 3 days after surgery, but tumor-associated NETs were not altered by intraoperative treatment.

*VATS* video-assisted thoracoscopic surgery

Simultaneously, the voltage-gated sodium channel Nav1.8 has been studied for its potential anti-cholinergic effects in the TME. An enhanced antitumor immunity was found after the use of lidocaine, a sodium channel blocker that targets voltage-gated sodium channels on nerve fibers and possesses anti-cholinergic effects.^516–518^ In a clinical trial examining the impact of lidocaine on 120 breast cancer patients (NCT02839668),^519^ these patients underwent surgical resection and received intravenous lidocaine as an anti-cancer treatment. It has been shown that lidocaine could reduce the release of MMP3, neutrophil extracellular traps (NETs), as well as myeloperoxidase, supporting that the recurrence rate would be reduced if lidocaine were administered for therapeutic purposes.^519^ An additional clinical trial (ChiCTR2000030629) corroborated the notion that lidocaine bolsters antitumor immunity, as evidenced by its effects in 95 patients with NSCLC.^520^ However, the clinical trial for pancreatic cancer, lidocaine did not alter overall or disease-free survival (NCT03245346).^521^ Lidocaine exhibited unfavorable effects in pancreatic cancer, and the underlying reasons may be that the cholinergic nervous system exerts an anti-pancreatic cancer effect, while lidocaine blocks cholinergic neurotransmission. Consequently, the use of lidocaine in pancreatic cancer may not yield favorable outcomes. More investigations are needed to elucidate the molecular mechanism.

### Clinical trials ongoing

A multitude of clinical trials focusing on neuroimmunotherapy are either in progress or have been completed but not yet published (Table 3). A substantial proportion of these trials explore the application of propranolol, expanding to a broader spectrum of tumor types, including gastrointestinal, prostate, and fallopian tube cancers. Nadolol, a nonselective β-AR blocker, is also under investigation in clinical studies for the treatment of infantile hemangiomas.^44,522^ Compared to propranolol, nadolol offers the advantage of a longer duration of action and greater convenience, potentially leading to its application in an even wider range of tumor types in the future. Donepezil, a drug utilized for the treatment of Alzheimer’s disease, inhibits AchE in the brain, thereby elevating Ach levels. Studies have applied donepezil to brain tumor, breast cancer, and head and neck cancer, with the aim of improving cognitive and emotional impairments following radiotherapy and chemotherapy in cancer patients. It is hypothesized that donepezil may reduce stress levels and enhance antitumor immunity. Additionally, bethanechol, a muscarinic agonist, is being investigated in clinical studies for pancreatic cancer. Investigators hypothesize that bethanechol treatment will alter nerve conduction within tumors by stimulating the parasympathetic nervous system, which may reduce tumor proliferation, macrophage activation, and decrease human cluster of CD44^+^ CSCs. Concurrently, the application of lidocaine has been expanded to more tumor types, such as colon and breast cancers.

**Table 3:** Ongoing clinical trials targeting neuro-immune axis in cancer

Drug	Targets	Highest phase	Cancer type	Identifier	Status
Propranolol	NE	II	Breast cancer	NCT02596867	Terminated
	NE	II	Prostate cancer	NCT05679193	Completed
	NE	II	Gastric cancer	NCT04005365	Unknown status
	NE	II	Prostate cancer	NCT01857817	Terminated
	NE	II	PADC	NCT06145074	Recruiting
	NE	II	Bladder cancer	NCT04493489	Unknown status
	NE	III	Colorectal cancer	NCT00888797	Unknown status
	NE	II	Colorectal metastasis	NCT03919461	Recruiting
	NE	I	NSCLC	NCT05979818	Not yet recruiting
	NE	I	Ovarian cancer	NCT01308944	Completed
	NE	II	Pancreatic cancer	NCT03838029	Recruiting
	NE	II	Kaposi sarcoma	NCT05797662	Not yet recruiting
	NE	II	Angiosarcoma	NCT04518124	Completed
	NE	II	Hepatic hemangioma	NCT03633747	Recruiting
	NE	II	Soft tissue sarcoma	NCT03108300	Unknown status
	NE	III	Melanoma	NCT02962947	Unknown status
	NE	I	Solid tumors	NCT02013492	Completed
	NE	Observational	Infantile hemangiomas	NCT04651049	Completed
	NE	II	Kaposi sarcoma	NCT06445166	Not yet recruiting
	NE	III	Infantile hemangioma	NCT01743885	Terminated
	NE	Observational	Infantile hemangioma	NCT01211080	Completed
	NE	II	Soft tissue sarcoma	NCT05961761	Recruiting
	NE	II	Breast cancer	NCT01847001	Completed
	NE	II	Prostate cancer	NCT03152786	Terminated
	NE	III	Infantile hemangioma	NCT02342275	Completed
	NE	IV	Spinal hemangioma	NCT05106179	Unknown status
	NE	IV	Pediatric hemangioma	NCT01908972	Completed
	NE	II	Melanoma	NCT03384836	Recruiting
	NE	II	Urothelial carcinoma	NCT04848519	Recruiting
	NE	I	Fallopian tube cancer	NCT01504126	Completed
	NE	II	Esophageal Adenocarcinoma	NCT04682158	Recruiting
	NE	II	TNBC	NCT05741164	Not yet recruiting
Nadolol	NE	II	Infantile Hemangiomas	NCT01010308	Completed
	Ach	III	Brain Tumors	NCT00369785	Completed
Donepezil	Ach	I	Brain Tumors	NCT00452868	Completed
	Ach	II	Breast Cancer	NCT01466270	Completed
	Ach	III	SCLC	NCT00006349	Completed
	Ach	III	Head and Neck Cancer	NCT00656513	Completed
	Ach	III	Head and Neck Cancer	NCT00003139	Completed
	Ach	III	Head and Neck Cancer	NCT00168181	Completed
	Ach	I	Pancreatic Cancer	NCT03572283	Active, not recruiting
Bethanechol	Ach	II	Pancreatic Cancer	NCT05241249	Recruiting
	Ionic channel	III	Breast Cancer	NCT01916317	Active, not recruiting
Lidocaine	Ionic channel	III	Colorectal cancer	NCT00236249	Completed
	Ionic channel	I	Colorectal Cancer	NCT02786329	Unknown Status
	Ionic channel	Not Applicable	Colorectal Cancer	NCT05250791	Recruiting
	Ionic channel	Not Applicable	Colorectal Cancer	NCT05484687	Completed
	Ionic channel	IV	Colorectal Cancer	NCT01841294	Unknown Status
	Ionic channel	Not Applicable	Pancreatic Cancer	NCT05470166	Completed
	Ionic channel	Not Applicable	Ovarian Cancer	NCT05450055	Not Yet Recruiting

In addition, there are numerous neuro-immune targets with the potential for further clinical research. For instance, drugs targeting CGRP, such as the monoclonal antibody Erenumab, have been commercialized and approved for the treatment of migraine. They hold significant promise in the treatment of tumors like metastatic melanoma, head and neck cancers, and pancreatic cancer,^124,127^ but their efficacy needs to be confirmed through well-designed clinical trials. Antidepressants targeting 5-HT have been shown to enhance antitumor immunity in mice,^153^ yet there is a lack of clinical trial evidence to support it. Modulating circadian rhythm genes and manipulating the brain-gut axis to strengthen antitumor immunity also have potential therapeutic value, warranting further investigations.

## Conclusion and future directions

Research on the interaction between the nervous and immune systems, especially regarding the neuro-immune-cancer axis (Table 4), remains limited. Neuronal components within TME hold significant research value, as they play a pivotal role in cancer. They modulate the immune response within TME by releasing neurotransmitters and neuropeptides, influencing the activity of immune cells. Pain and symptoms associated with cancer may be related to the activation of neuronal components, and understanding these mechanisms could aid in the development of more effective pain management strategies. Neuronal components may also affect the tumor response to therapies, including chemotherapy, radiotherapy, and immunotherapy. The presence of neural components or specific neuronal markers may serve as biomarkers for predicting tumor prognosis and therapeutic responses. Investigating neural components in TME may reveal new drug targets, opening avenues for targeted therapeutic strategies. Furthermore, these components are associated with tumor invasiveness and metastasis, and understanding this relationship could aid in developing strategies to prevent tumor spread. Examining how tumors affect the nervous system may inform strategies to minimize neurological damage during cancer treatment.

**Table 4:** Cancer types that involve in neuro-immune crosstalk

Cancer types	Immune components	Neural components	Key molecules in pathogenesis
Glioma	TAM	Glioma cell	IL-10, EGF, VEGF^76–78^
	T cell	Glioma cell	PD-L1, TGF-β, CCL5^96,97^
	B cell	Glioma cell	PD-L1, TGF-β, IL-10^103–105^
	NK cell	Glioma cell	IL-2, IFN-α, TGF-β^86,90,107^
	DC	Glioma cell	TSP-1, IL-4, IL-5^107^
	MDSC	Glioma cell	PD-L1, IL-10, TGF-β^106^
	microglia	Neuron	Midkine, CCL4, CCL5^108^
HNSCC	TILs	Trigeminal nerve	CGRP- RAMP1^123,124^
	PD-L1	Trigeminal nerve	GDNF, PD-L1, JAK2, STAT1^122^
Oral cancer	CD4^+^ and CD8^+^ T lymphocytes NK cells	Sensory nerve	CGRP- RAMP1^168^
Melanoma	CD8^+^ T cells	Sensory nerve	TRPV1, CGRP, RAMP1^127^
	Macrophages	Schwann cells	IL-6, MIF, FGF2, VEGF^165^
	DCs	stress	DA, DRD3^303^
Pancreatic cancer	T cells	Sympathetic nerve	MHC I, B7-1, B7H-1^141^
	mAchR^+^ Macrophages	parasympathetic nerve	TNF-α^147^
	PD-L1	5-HT	Histone serotonylation, PD-L1^153^
B cell lymphoma	CD8^+^ T cells	Sympathetic nerve	IFN-γ^142^
Cervical Cancer	CD8^+^ T cells	Sympathetic nerve	β2-AR^136^
	CCL2	Schwann cells DRG	CCL2, CCR2, MMP2, MMP9, MMP12^166^
CRC	Tm	Parasympathetic nerve	TFF2, IL-17A, IL-1β^150^
	MDSCs	GABA	GABA, GABAAR^151^
	CD8^+^ T cells	GABA	GABA, GABABR^152^
	TRM	DA	DA transporter, G9a^156^
	CD8^+^ T cells	DA	DRD5, CD103^155^
NSCLC	CD8^+^ T cells	stress	GCs, TSC22D3^282^
	Monocytes, T cells	stress	DA, DRD1^303^
Gastric cancer	PD-L1	5-HT	Histone serotonylation, PD-L1^153^
Breast cancer	TAM T cells	HA	HRH1, VISTA^160^
	Tregs	HA	HRH4^161^
	Immunological synapse	NAA	PCAF, laminA-K542^163^
	Neutrophils	stress	GCs, NETs^290^
	Monocytes	stress	GCs, TSC22D3^282^
SCLC	CD8^+^ T cells	SCLC cells	PD-1, CTLA-4, Tim3^180^
Pan-NENs	T cell	Pan-NENs cells	PD-L1^182^
MTC	DCs	Sensory nerve	CGRP, CALCRL, RAMP1^128^
Prostate cancer	TAMs	stress	NE, NPY^313^

*TSP-1* thrombospondin-1, *MIF* macrophage migration inhibitory factor, *Tm* memory *T cell*, *TRM* tissue-resident memory T cells*, HRH1* histamine receptor H1, *VISTA* V-domain immunoglobulin suppressor of T cell activation, *PCAF* p300/CBP-associated factor, *Tim3* T cell immunoglobulin mucin 3, *LAG3* lymphocyte activation gene-3, *LAYN* layilin, *MTC* medullary thyroid carcinoma, *NPY* neuropeptide Y

Targeting the neuro-immune-cancer axis may emerge as a more comprehensive, holistic, and effective novel approach to cancer treatment. Elucidating the molecular mechanisms of them in TME, including neurotransmitters, cytokines, receptors, and signaling pathways, is crucial for precise tumor therapy. Depending on the role of neural components in different TME, selectively enhancing or blocking neural transmission may augment the efficacy of existing antitumor treatments. Systemic stress, stress disorders, mood disorders, and circadian rhythm disruptions can profoundly impact the homeostasis of the immune system, the growth of tumors, and the response to treatment. The appropriate application of existing medications such as sedatives, anti-anxiety and antidepressant drugs, and anti-epileptic drugs combined with antitumor therapy may improve patients’ quality of life and extend survival time.

Studying the neuro-immune-cancer axis will face numerous challenges in the future: both the nervous and immune systems consist of a vast array of different cell types, each with distinct functions and interactions. Distinguishing and investigating these cell types requires highly specialized techniques. Neuro-immune interactions involve complex molecular mechanisms. Unraveling these mechanisms necessitates precise molecular biology tools. Neuro-immune interactions may have different functions and effects at various times and places. Studying this spatiotemporal control requires meticulous experimental design and analytical methods. The interplay between the nervous and immune systems may interact with other systems (such as the endocrine system), and studying these multi-system interactions requires interdisciplinary approaches. Although animal models are vital tools for this field, they may not fully replicate the conditions of human cancers. The data generated from neuro-immune research is voluminous and complex, necessitating sophisticated bioinformatics tools and statistical methods for processing and interpretation.

Overcoming technical hurdles in studying neuro-immune interactions in TME demands collaboration across neuroscience, immunology, oncology, pathology, and pharmacology. It involves innovative designs, advanced techniques, and strong data analysis. As science progresses, these challenges are being met, leading to deeper insights into neuro-immune-cancer dynamics. Continued interdisciplinary efforts are key to developing new cancer therapies targeting these interactions.
